# A Jurassic pterosaur from Patagonia and the origin of the pterodactyloid neurocranium

**DOI:** 10.7717/peerj.2311

**Published:** 2016-08-30

**Authors:** Laura Codorniú, Ariana Paulina Carabajal, Diego Pol, David Unwin, Oliver W.M. Rauhut

**Affiliations:** 1 Departamento de Geología, CONICET, Universidad Nacional de San Luis, San Luis, Argentina; 2 Instituto de Investigaciones en Biodiversidad y Medioambiente (INIBIOMA), CONICET, Río Negro, Argentina; 3 CONICET, Museo Paleontológico Egidio Feruglio, Trelew, Chubut, Argentina; 4 School of Museum Studies, University of Leicester, Leicester, United Kingdom; 5 Department of Earth and Environmental Sciences and GeoBioCenter, Bayerische Staatssammlung für Paläontologie und Geologie, Munich, Germany

**Keywords:** Pterosauria, Cañadón Asfalto, Patagonia, Chubut, Middle Jurassic

## Abstract

Pterosaurs are an extinct group of highly modified flying reptiles that thrived during the Mesozoic. This group has unique and remarkable skeletal adaptations to powered flight, including pneumatic bones and an elongate digit IV supporting a wing-membrane. Two major body plans have traditionally been recognized: the primitive, primarily long-tailed paraphyletic “rhamphorhynchoids” (preferably currently recognized as non-pterodactyloids) and the derived short-tailed pterodactyloids. These two groups differ considerably in their general anatomy and also exhibit a remarkably different neuroanatomy and inferred head posture, which has been linked to different lifestyles and behaviours and improved flying capabilities in these reptiles. Pterosaur neuroanatomy, is known from just a few three-dimensionally preserved braincases of non-pterodactyloids (as Rhamphorhynchidae) and pterodactyloids, between which there is a large morphological gap. Here we report on a new Jurassic pterosaur from Argentina, *Allkaruen koi* gen. et sp. nov., remains of which include a superbly preserved, uncrushed braincase that sheds light on the origins of the highly derived neuroanatomy of pterodactyloids and their close relatives. A µCT ray-generated virtual endocast shows that the new pterosaur exhibits a mosaic of plesiomorphic and derived traits of the inner ear and neuroanatomy that fills an important gap between those of non-monofenestratan breviquartossans (Rhamphorhynchidae) and derived pterodactyloids. These results suggest that, while modularity may play an important role at one anatomical level, at a finer level the evolution of structures within a module may follow a mosaic pattern.

## Introduction

Pterosaurs first appeared in the Late Triassic and went on to achieve high levels of morphologic and taxonomic diversity during the Mesozoic, with more than 150 species recognized so far (Barrett et al., 2008; Butler, Benson & Barrett, 2013; Foth, Brusatte & Butler, 2012; Witton, 2013; Benson et al., 2014). They have traditionally been divided into two major groups, “rhamphorhynchoids” (a paraphyletic assemblage of basal pterosaurs) and pterodactyloids (Plieninger, 1901). “Rhamphorhynchoids” (Unwin, 2003) now generally referred to as non-pterodactyloids (Bell & Padian, 1995), are characterized by a long tail (except for some anurognathids) and short neck and metacarpus, whereas pterodactyloids have a much larger body size range, an elongated neck and metacarpus, and a relatively short tail. Furthermore, the endocranium of pterodactyloids (Edinger, 1941; Klinghardt, 1941; Wellnhofer, 1970; Lewy, Milner & Patterson, 1992; Kellner, 1996; Lü et al., 1997; Bennett, 2001; Witmer et al., 2003; Eck, Elgin & Frey, 2011) is strongly modified compared to that of non-pterodactyloids (Newton, 1888; Edinger, 1927; Wellnhofer, 1975; Witmer et al., 2003; Gasparini, Fernández & De la Fuente, 2004). Thus, in contrast to “rhamphorhynchoids” the cerebral hemispheres are enlarged, the pontine flexure is pronounced, and the optic lobes are located beneath the cerebral hemispheres, mimicking the neuroanatomy of birds in several respects. These modifications have important implications for behaviour and sensory functions (Witmer et al., 2003).

Recently discovered taxa such as *Darwinopterus* from the early Late Jurassic of China (Wang et al., 2009; Wang et al., 2010; Lü et al., 2010; Liu et al., 2012; Sullivan et al., 2014) appear to represent a transitionary stage that partially fills the morphological gap between “rhamphorhynchoids” and pterodactyloids (Lü et al., 2010; Andres, Clark & Xu, 2014). *Darwinopterus* and its sister taxon, Pterodactyloidea, form a clade, Monofenestrata diagnosed by the confluence of the narial and antorbital opening in a single fenestra (Lü et al., 2010). *Darwinopterus* combines a “rhamphorhynchoid” body with a pterodactyloid neck and skull, hinting at a modular type of evolution (Lü et al., 2010). *Darwinopterus* and other basal monofenestratans (Wang et al., 2009; Wang et al., 2010; Lü et al., 2010; Liu et al., 2012; Sullivan et al., 2014) are, however, known only from compressed and semi-compressed remains that yield limited braincase and endocranial information.

Here we describe a new pterosaur that is represented by several skeletal elements including an almost perfect, three-dimensionally preserved braincase that shows a unique combination of characters shared with both non-monofenestratan breviquartossans (Rhamphorhynchidae sensu Witton, 2013) and pterodactyloids. Recovered by phylogenetic analysis as the sister group of Monofenestrata, details of this pterosaur provide insights into the origin of the pterodactyloid neurocranium and improve our understanding of the tempo and mode of pterosaur evolution.

## Geological Setting

The type and referred material of the new taxon comes from a single locality within the Cañadón Asfalto Formation in northern central Chubut Province, Argentina. The Cañadón Asfalto Formation is a predominantly lacustrine unit mainly composed of shales, lacustrine limestones and frequent conglomeratic and tuffacious intercalations (Stipanicic et al., 1967; Tasch & Volkheimer, 1970; Cabaleri et al., 2010). The locality is placed in the Canadón Carrizal, in a section of thin-bedded and strongly silicified lacustrine limestones, probably representing a pan lake (Cabaleri, Armella & Silva Nieto, 2005). The pterosaur bones were found in an extensive bonebed in the upper 5 cm of a limestone bed with a thickness of 15–20 cm; the bonebed can be followed laterally for at least 30 m and might be more extensive. The remains are usually disarticulated, though some association of different elements is present, and one wing digit and two sets of mandibles were found partially articulated (Codorniú, Rauhut & Pol, 2010). The holotype braincase MPEF-PV 3613 was found in close association with the cervical vertebra MPEF-PV 3616 and the mandibles MPEF-PV 3609, and these remains probably represent a single individual. The cervical vertebra MPEF-PV 3615 is identical to cervical MPEF-PV 3616 in all discernable characters and is thus also referred to the same taxon. However, given the possibility that more than a single taxon is present in the locality, the referral of all of this material should be regarded as tentative. To establish the phylogenetic relationships of the new taxon we analyzed a matrix in which we restricted information to the braincase alone, and then conducted further analyses that included information from both the holotype and the referred specimens (see below). That both analyses, with only the braincase and with the addition of all known material, result in trees with the same topology further supports the association of these materials.

The age of the Cañadón Asfalto Formation was, until recently, usually given as Callovian-Oxfordian (latest Middle to earliest Late Jurassic), but new radiometric and biostratigraphic evidence indicates that the formation is considerably older, with ages ranging from the Toarcian (latest Early Jurassic; (Cúneo et al., 2013) to the earliest Bathonian (Volkheimer et al., 2008; Cabaleri et al., 2010; Cúneo et al., 2013). For a more thorough discussion of the age of the Cañadón Asfalto Formation, see Cúneo et al. (2013).

## Methods

### CT-scan data

CT scans of the braincase (MPEF-PV 3613) were made by scanner v|tome|x s (GE Sensing & Inspection Technologies GmbH phoenix|X-ray). The images (720 coronal stacks) have a 1024 pixel resolution and a voxel size of 0.047310 × 0.047310 × 0.047310 mm. Virtual three-dimensional reconstructions of the braincase, cranial endocast and inner ear were generated using the software Materialise Mimics (10.0) at the University of Alberta Paleovertebrate Laboratory; and the resulting 3D models were then imported into the software Geomagic (10.0). Illustrations were made using Adobe Photoshop (C). The terminology used for braincase pneumatic cavities follows Witmer (1997) and Dufeau (2011).

### Nomenclatural acts

The electronic version of this article in Portable Document Format (PDF) will represent a published work according to the International Commission on Zoological Nomenclature (ICZN), and hence the new names contained in the electronic version are effectively published under that Code from the electronic edition alone. This published work and the nomenclatural acts it contains have been registered in ZooBank, the online registration system for the ICZN. The ZooBank LSIDs (Life Science Identifiers) can be resolved and the associated information viewed through any standard web browser by appending the LSID to the prefix “http://zoobank.org/”. The LSID for this publication is: urn:lsid:zoobank.org:pub:48910653-0343-4A8D-911F-3498A755F305. The online version of this work is archived and available from the following digital repositories: PeerJ, PubMed Central and CLOCKSS.

### Phylogenetic analysis

#### Taxon and character sampling

The data matrix used in the phylogenetic analysis is based on a revision of a previously published phylogenetic analysis (Lü et al., 2010), with the addition of six new characters derived from the skull region (most of which are focused on braincase anatomy) and one character proposed by Andres & Ji (2008). Seven characters of the original dataset have been modified (see SI., Character List) and three characters (25, 78, and 112) have been excluded from this dataset given that they had vague character state definitions that did not fit the analysed morphological diversity. The taxon sampling was also expanded from the original dataset by including a recently described basal monofenestratan pterosaur from China: *Wukongopterus* (Wang et al., 2009). Furthermore, the hypothetical ancestor used by Lü et al. (2010) was replaced by codings for two outgroup representatives, the basal archosauriform *Euparkeria* and the basal dinosaur *Herrerasaurus*. The resultant data matrix includes 59 taxa scored across 123 characters. One of the multistate characters was treated as ordered whereas the rest of the characters were treated as unordered (see SI., Character List).

Two different phylogenetic analyses were conducted. First, the character codings for *Allkaruen koi* were restricted to the anatomical information provided by the braincase (MPEF-PV 3613) to test the phylogenetic information provided by this material. Second, a more inclusive phylogenetic analysis was conducted scoring all the known materials (MPEF-PV 3609, mandible; MPEF-PV 3615, 1616; cervical vertebrae) to determine the phylogenetic position of the new taxon.

#### Phylogenetic analyses and results

The analyses were conducted under equally weighted parsimony in TNT (Goloboff, Farris & Nixon, 2008) through a heuristic search of 1,000 replicates of Wagner trees followed by TBR branch swapping.

The analysis of the dataset with the character scorings of only the braincase of *Allkaruen koi* resulted in 360 most parsimonious trees (using the collapsing rule 3 for zero-length branches; see Coddington & Scharff, 1994). These trees have 413 steps (CI = 0.419, RI = 0.791). In all most parsimonious trees *Allkaruen* is located as the sister taxon to Monofenestrata, a clade consisting of Pterodactyloidea, *Darwinopterus*, and *Wukongopterus*. Analysis of the dataset with character scorings for all the known materials of *Allkaruen koi* resulted in the same 360 most parsimonious trees (using the collapsing rule 3 for zero-length branches; see Coddington & Scharff, 1994). These trees have 416 steps (CI = 0.416, RI = 0.789). The same phylogenetic position was retrieved for *Allkaruen*, as the sister taxon of Monofenestrata [Pterodactyloidea + (*Darwinopterus* + *Wukengopterus*)]. The following discussion of consensus trees, support measures, and synapomorphy lists are based on the dataset with the most inclusive scoring of *Allkaruen koi* (support values when only the skull scores for *Allkaruen* were included, were also calculated and are shown below).

## Results

### Systematic paleontology

**Table:** 

Pterosauria Kaup, 1834
Breviquartossa Unwin, 2003
*Allkaruen koi* gen. et sp. nov.

**Zoobank.** urn:lsid:zoobank.org:act:C545BD35-B448-4D47-A2A6-14215E9E3155

**Etymology**. Genus name from the native Tehuelche word ‘*all*’ meaning brain, and ‘*karuen*,’ meaning ancient. Species name from Tehuelche ‘*koi*’ meaning lake, referring to the lacustrine setting of the type locality.

**Holotype**. MPEF-PV (Museo Paleontológico Egidio Feruglio) 3613, braincase, a mandible (MPEF-PV 3609) and a cervical vertebrae (MPEF-PV 3615) (Figs. 1–7; Figs. S1–S3).

**Referred material**. Referred material includes a mid cervical vertebrae (MPEF-PV -3616.

**Type locality and horizon**. Locality La Lluvia, Cañadón Carrizal, Cerro Cóndor, Chubut, Argentina. Cañadón Asfalto Formation, latest Early-early Middle Jurassic (Cúneo et al., 2013).

**Diagnosis**. Small pterosaur diagnosed by the following unique combination of skull characters present in the holotype (autapomorphies marked with asterisk): frontal with large pneumatic foramen on the postorbital process; dorsal occiput faces posterodorsally and occipital condyle faces posteroventrally; long, rod-like basipterygoid processes diverging at approximately 20–25 degrees. The referred mandibular and vertebral materials also show a unique combination of characters that include a long lower jaw with a concave profile in lateral view; four-five large, septated, and well-separated anterior alveoli followed by a posterior alveolar groove*; mid-cervical vertebrae elongate with low neural arch and blade-like neural spine; pneumatic foramina on lateral surface of the centrum and peduncle of the neural arch; reduced diapophyseal process lacking articular surface; absence of accessory zygapophyseal processes.

**Figure 1:**
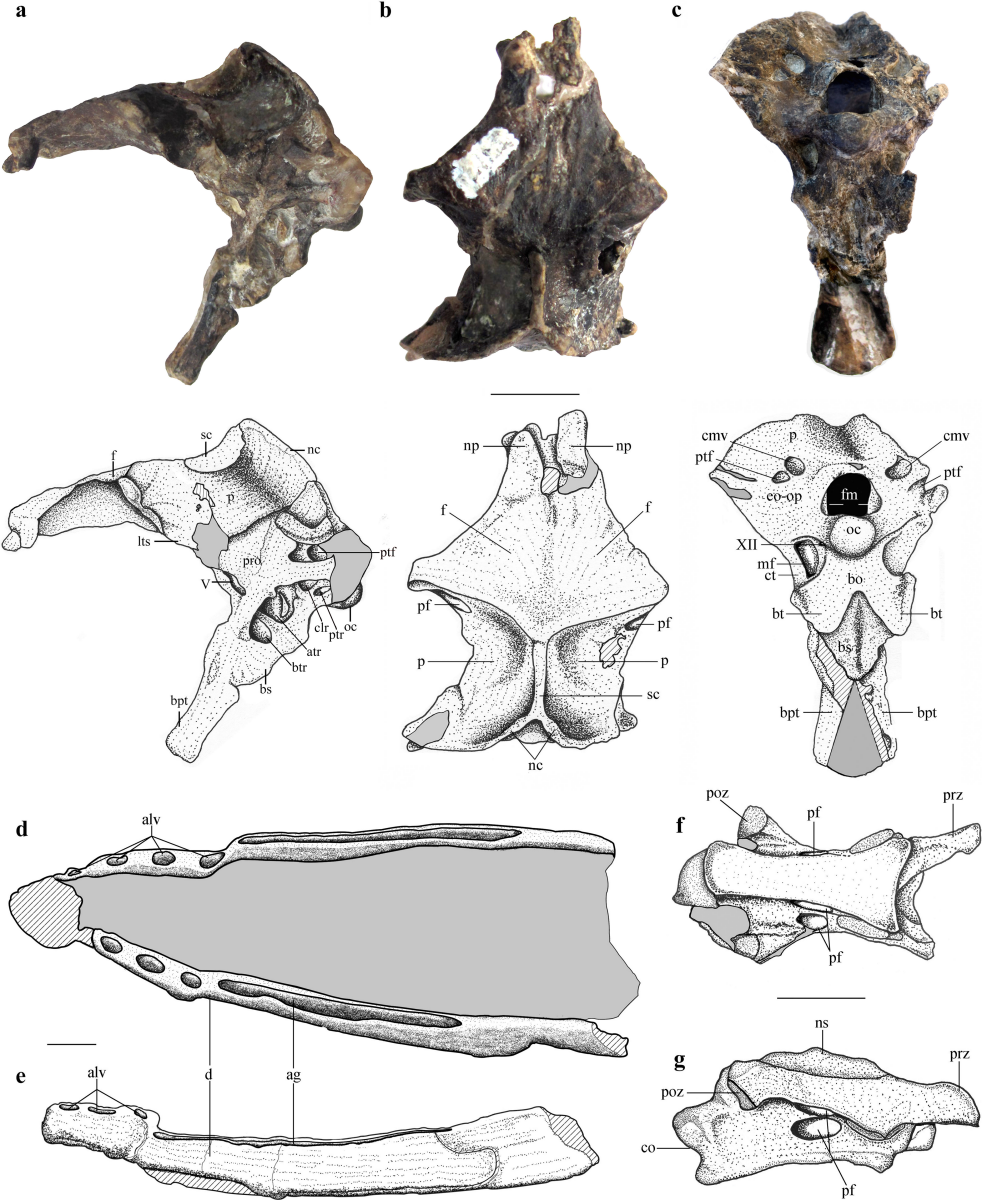
Selected skeletal elements of *Allkaruen koi*. (A–C), Holotype braincase (MPEF-PV 3613) in left lateral (A), dorsal (B) and posteroventral (C) views. (D, E), Mandible (MPEF-PV 3609) in dorsal (D) and right lateral (E) view. (F, G), Cervical vertebra (MPEF-PV 3615) in ventral (F) and right lateral (G) view. Abbreviations: ag, alveolar groove; alv, alveoli; atr, anterior tympanic recess; bo, basioccipital; btr, basipterygoid recess; bpt, basipterygoid process; bs, basisphenoid; bt, basal tuber; cmv, caudal middle cerebral vein foramen; co, condyle; ct, crista tuberalis; d, dentary; eo-op, exoccipital/opisthotic; f, frontal; fm, foramen magnum; lts, laterosphenoid; mf, metotic foramen; nc, nuchal crest; np, nasal process; ns, neural spine; oc, occipital condyle; p, parietal; pf, pneumatic foramen; poz, postzygapophysis; pro, prootic; prz, prezygapophysis; ptf, posttemporal fenestra; ptr, posterior tympanic recess; sc, sagittal crest. Roman numerals indicate cranial nerves. Scale bars are 10 mm.

**Figure 2:**
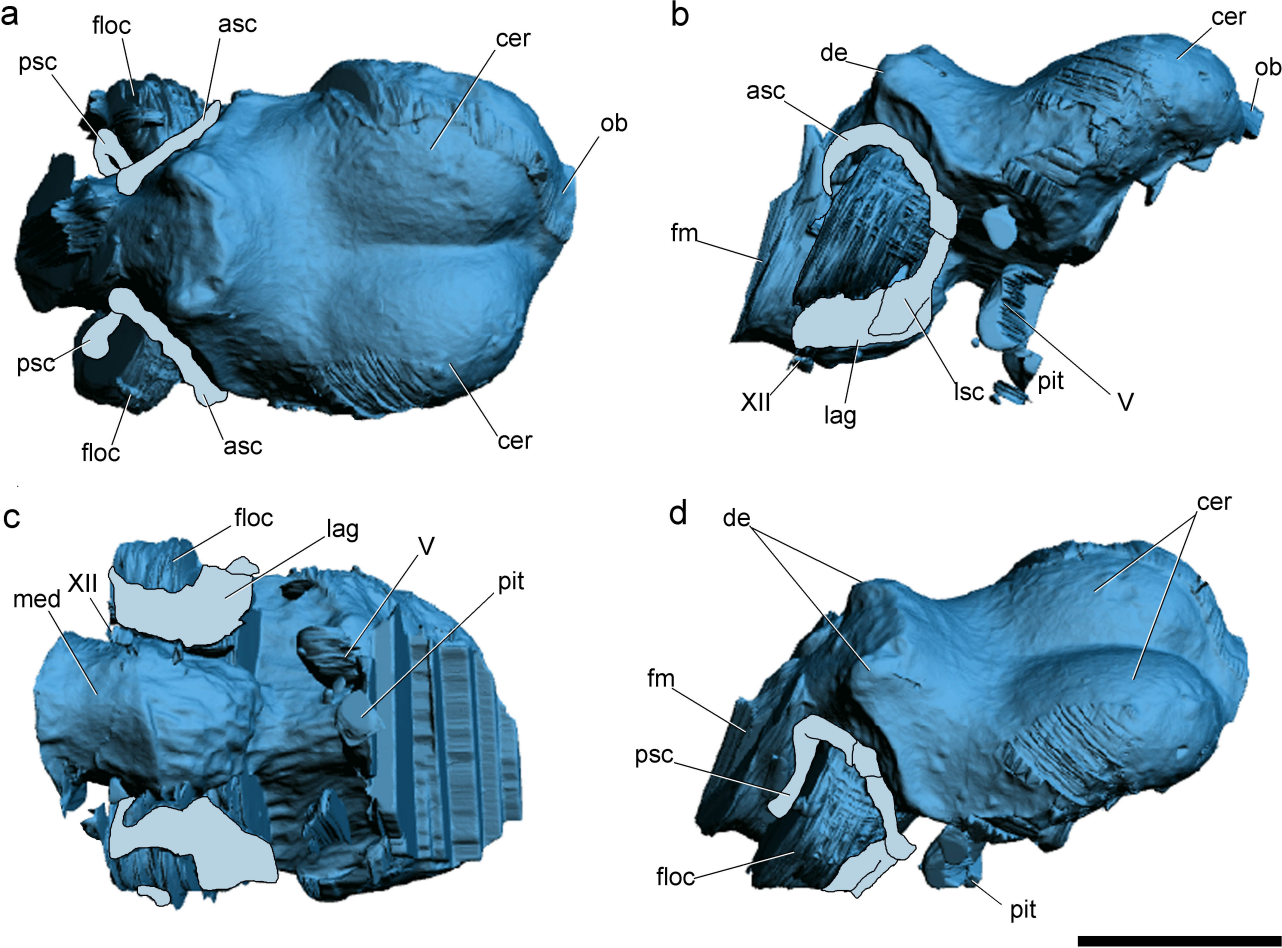
Surface-rendered CT-based reconstructions of the cranial endocast and endosseous labyrinth of the holotype of *Allkaruen koi,* in dorsal (A), right lateral (B), ventral (C) and dorsolateral (D) views. Abbreviations: asc, anterior semicircular canal; cer, cerebral hemisphere; de, dorsal expansion ; floc, flocculus; fm, foramen magnum; lag, lagena; med, medulla oblongata; lsc, lateral semicircular canal; ob, olfactory bulb; pit, pituitary body; psc, posterior semicircular canal; V, XII, cranial nerves. Scale bar is 1 cm.

**Figure 3:**
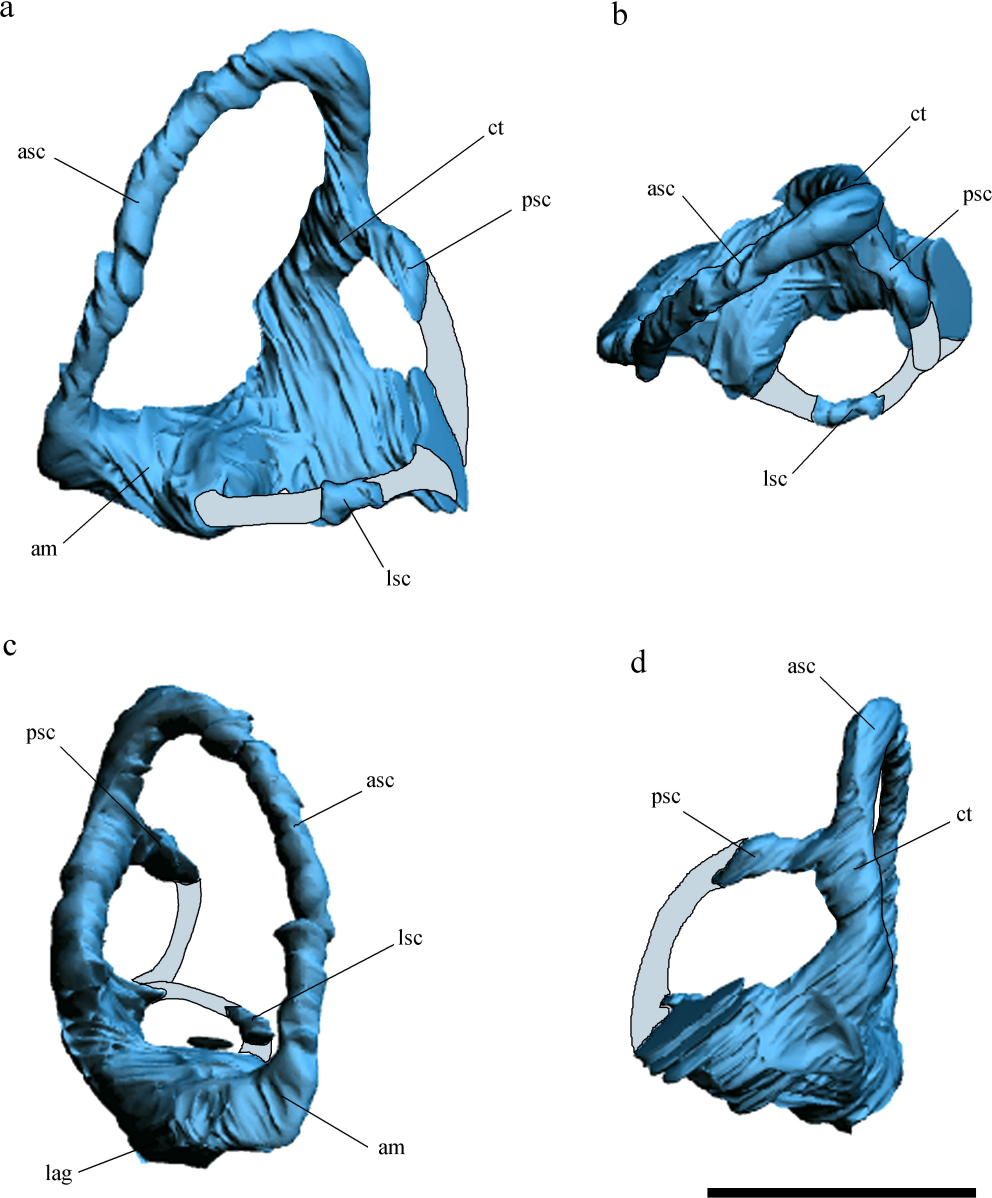
Inner ear anatomy. Digital reconstruction of the left inner ear of *Allkaruen koi,* based on the CT scan of the holotype in lateral (A), dorsal (B), anterior (C) and posteromedial (D) views. Reconstructed sections are in light-blue. Abbreviations: am, anterior ampula; asc, anterior semicircular canal; ct, common trunk; lag, lagena; lsc, lateral semicircular canal; psc, posterior semicircular canal. Scale bar is 0.5 cm.

**Figure 4:**
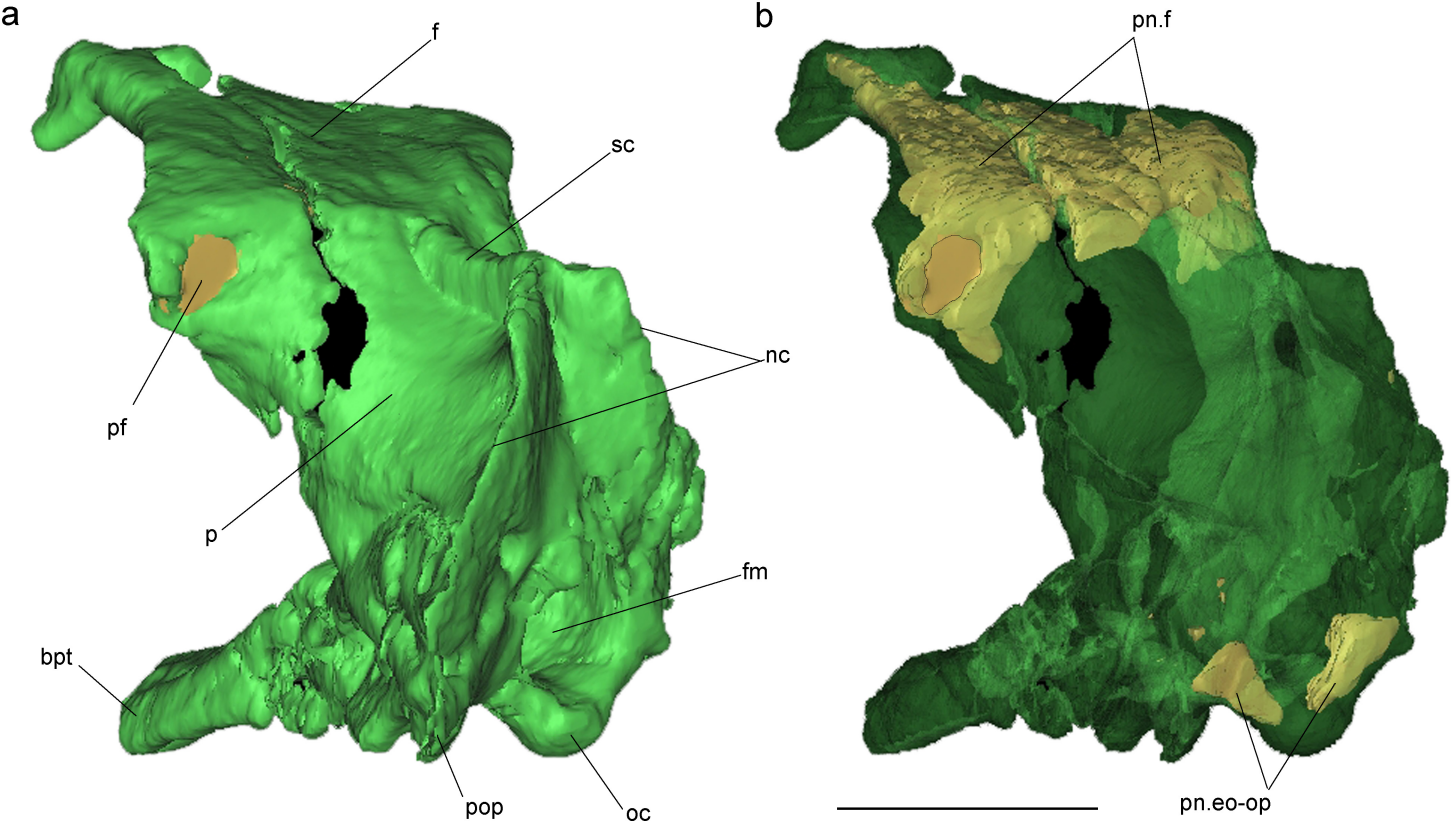
Volume-rendered CT-based reconstruction of the braincase of the holotype of *Allkaruen koi,* in left laterodorsal view. The bone is rendered solid (A), and semi-transparent (B). Pneumatic recesses are shown in yellow in (B). Abbreviations: bpt, basipterygoid process; f, frontal; fm, foramen magnum; nc, nuchal crest; oc, occipital condyle; p, parietal; pf, pneumatic foramen; pop, paroccipital process; pn.f, frontal pneumaticity; pn.eo-op, exoccipital-opisthotic pneumaticity; sc, sagittal crest. Scale bar is 1 cm.

**Figure 5:**
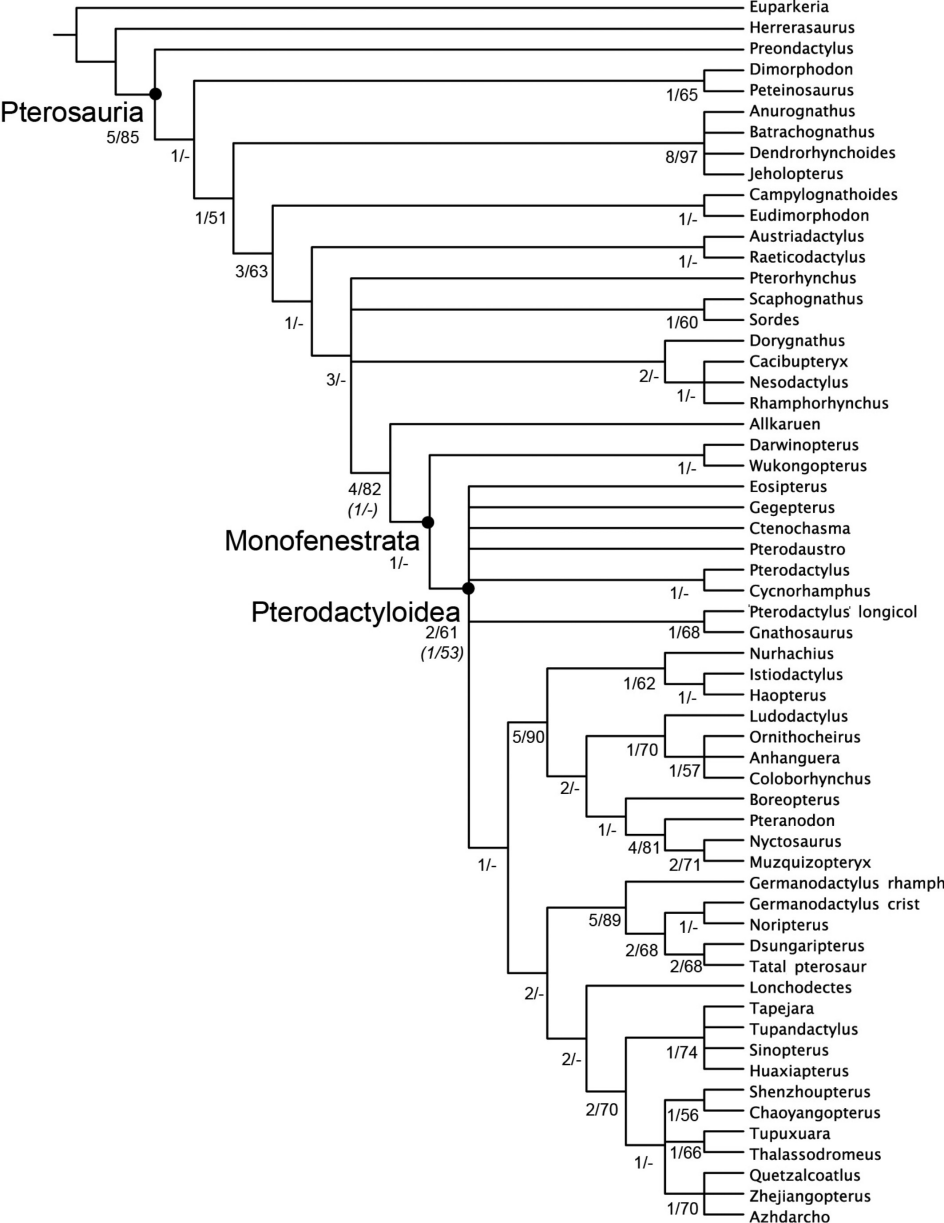
Strict consensus of the 360 most parsimonious trees with major nodes of Pterosauria labelled. Numbers at the nodes represent the Bremer support value and Bootstrap frequencies for each of the nodes present in the strict consensus tree.

**Figure 6:**
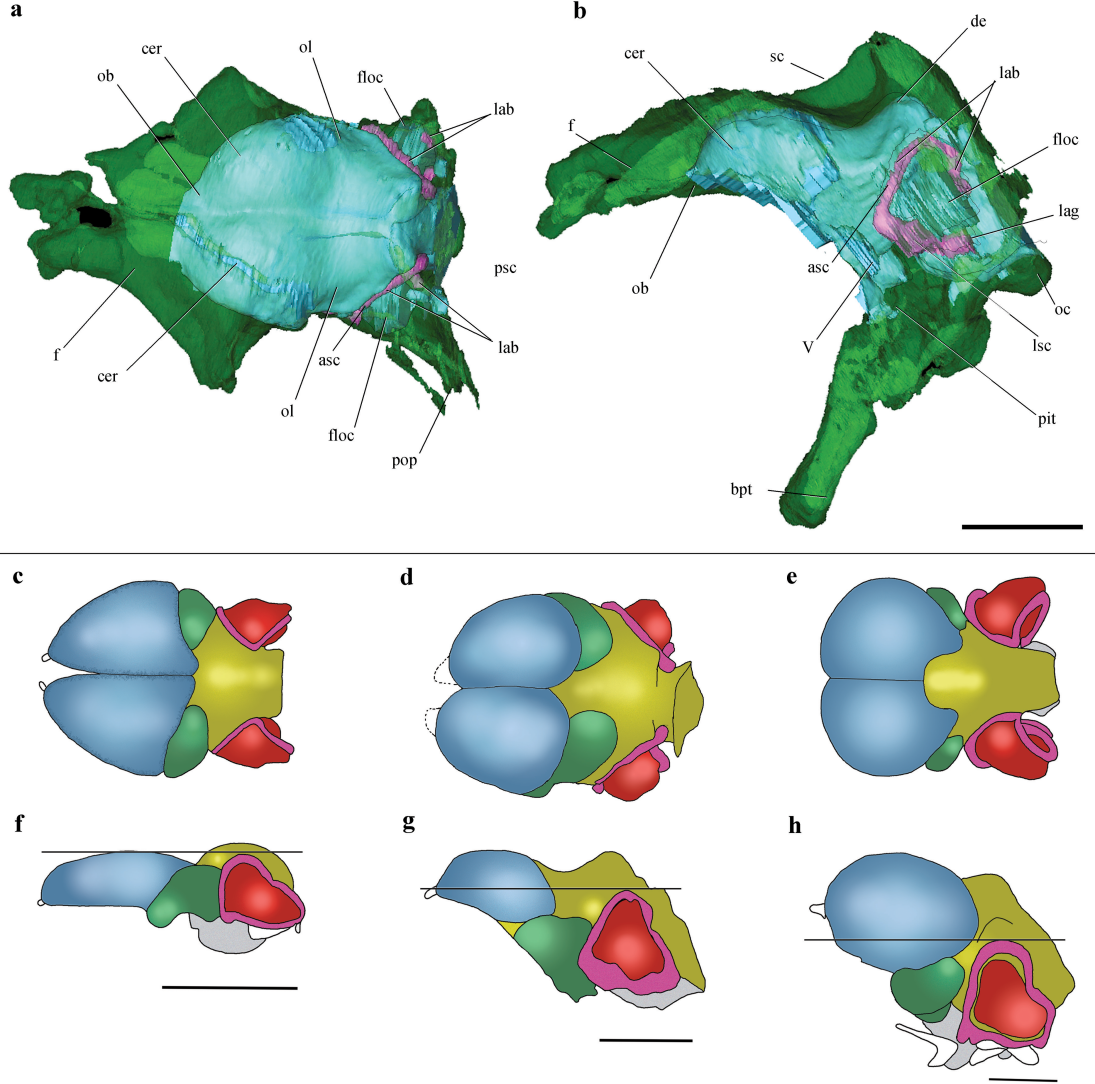
Cranial endocast and comparison of brain anatomy in pterosaurs. (A, B), Volume-rendered CT-based reconstruction of the braincase of the holotype of *Allkaruen koi*, in dorsal (A) and left lateral (B) views (the bone is rendered semitransparent to show the cranial endocast and the inner ear). (C–H), schematic drawings of brain anatomy in *Rhamphorhynchus* (C, F), *Allkaruen* (D, G), and *Anhanguera* (E, H) in dorsal (C–E) and lateral (F–H) views. Colors (C–H) indicate equivalent brain regions (blue, cerebrum; green, optic lobe; yellow, cerebellum; red, floccular process of cerebellum; pink, semicircular canals). The horizontal black line shows the relationship between the dorsal expansion of the anterior semicircular canal and the forebrain. Abbreviations: asc, anterior semicircular canal; cer, cerebral hemisphere; de, dorsal expansion; f, frontal; floc, floccular process of cerebellum; lab, labyrinth of inner ear; lsc, lateral semicircular canal; lag, lagena; ob, olfactory bulb; oc, occipital condyle; ol, optic lobe; pbt, basipterygoid process; pit, pituitary body; psc, posterior semicircular canal; sc, sagittal crest. Roman numerals indicate cranial nerves. Brain anatomy of *Rhamphorhynchus* and *Anhanguera* modified from Witmer et al. (2003). Scale bars are 10 mm.

**Figure 7:**
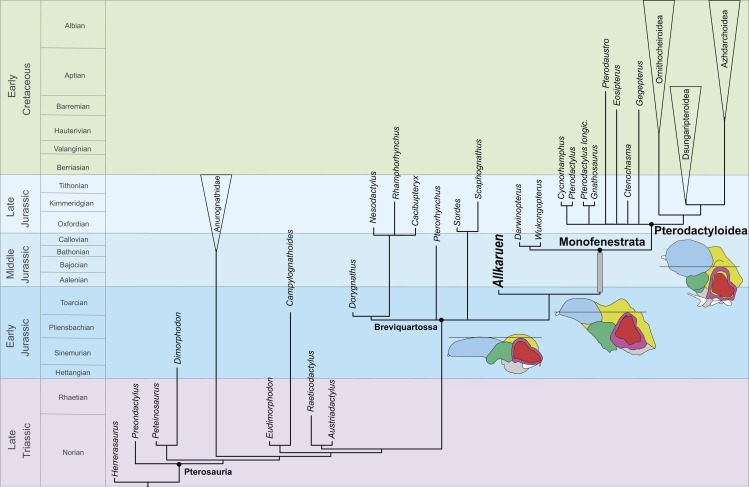
Phylogenetic position of *Allkaruen koi* calibrated against geological time. The topology is based on the strict consensus and summarizes the relationships among basal pterosaurs (“rhamphorhynchoids”) and major groups of Pterodactyloidea (collapsed into clades). Grey bar represents ghost lineage leading to Monofenestrata and schematic endocast drawings represent the condition of “rhamphorhynchoids,” *Allkaruen*, and pterodactyloids. Further phylogenetic information is given in the Supplemental Materials.

### Description

The braincase is undistorted and superbly preserved. All skull bones are fused, as in most osteologically mature pterosaurs. In the skull roof, frontals and parietals are completely fused with each other and the rest of the braincase. The parietals are long (60% of the frontal length, excluding the nasal processes), as seen in *Scaphognathus* (Geol. Paläont. Inst. Univ. Bonn, Nr. 1304) and *Campylognathoides* (Padian, 2008: Plate 5, Fig. 1), but unlike the relatively shorter parietal observed in other pterosaurs, such as *Rhamphorhynchus* (Wellnhofer, 1975), *Cacibupteryx* (Gasparini, Fernández & De la Fuente, 2004), *Parapsicephalus* (Newton, 1888), *Pteranodon* (Bennett, 2001) and *Anhanguera* (Kellner & Tomida, 2000). The supratemporal fossae are deeply excavated on the parietals, separated by a low and narrow sagittal crest (Fig. 1). In dorsal view, this crest is bifurcated at the occipital margin of the skull roof, leading to a V-shaped nuchal crest. The dorsal surface of the frontals is broad and flat. It is 22.5 mm in total length (13 mm long excluding the nasal process). The nasal processes are slightly separated by a narrow gap which originally accommodated the caudal extension of the premaxillae. That the latter are absent suggests that the premaxillae and frontals had not yet fused. The postorbital process of the frontal projects strongly laterally. The dorsal surface of the frontals curves abruptly ventrally towards the posterior end of the postorbital processes to form the anterior margin of the supratemporal fossa. Each frontal has a large oval foramen on the posterior surface of the postorbital process (Figs. 1B and 4), within the margin of the supratemporal fossa, which communicates internally with a large and complex pneumatic cavity that pneumatizes almost the entire frontal, as shown by the CT scans.

The occiput is trapezoidal in shape, slightly concave transversely, and faces posteriorly, resembling the morphology of *Cacibupteryx* (Gasparini, Fernández & De la Fuente, 2004) and *Rhamphorhynchus* (Wellnhofer, 1978) (Fig. 1C). The occipital region in pterodactyloids (e.g., *Anhanguera*, *Pteranodon*, *Tapejara*) is posteroventrally deflected with respect to the longitudinal axis of the skull and is oval in outline, with rounded lateral margins (Wellnhofer, 1985; Wellnhofer & Kellner, 1991; Bennett, 2001). The supraoccipital makes up more than half the height of the occipital plane (14.5 mm of a total height of 24 mm) above the foramen magnum. It is mostly flat lacking a backward extension, and becomes gradually concave dorsally. Just dorsal and lateral to the foramen magnum there is a large, high oval to kidney-shaped opening corresponding to the caudal middle cerebral vein foramen. Ventrolateral to this foramen, there is a second, smaller opening corresponding to the postemporal fenestra, which is enclosed between the supraoccipital and parietal. In *Allkaruen* the postemporal fenestra is slightly smaller to subequal in size to the caudal middle cerebral vein foramen, as in *Rhamphorhynchus* (Wellnhofer, 1975). This contrasts with the condition of pterodactyloid pterosaurs, such as *Anhanguera piscator* (Kellner & Tomida, 2000), *Anhanguera santanae* (Wellnhofer, 1985), *Tapejara wellnhoferi* (Wellnhofer & Kellner, 1991; Kellner, 1996), and *Pteranodon* (Bennett, 2001), in which the postemporal fenestra is almost twice the size of the exit of the dorsal head vein.

The occipital condyle is posteroventrally directed with respect to the longitudinal axis of the skull, and thus also angled in respect to the plane of the dorsal part of the occiput. This is comparable to the condition in monofenestratans including basal forms such as *Darwinopterus* (D Unwin, 2016, unpublished data), but differs from the construction in basal pterosaurs, for example *Rhamphorhynchus* (Witmer et al., 2003) where the occipital condyle is parallel to the long axis of the skull. The occipital condyle is much smaller than the foramen magnum, as in *Rhamphorhynchus*, *Cacibupteryx*, *Rhamphinion*, and *Tapejara*, but unlike *Pteranodon* and *Anhanguera* in which the occipital condyle is larger. The foramen magnum in *Allkaruen* is large, oval in shape and higher (7 mm) than wide (5.5 mm). Laterally the exoccipital contacts the opisthotic, but the suture is obliterated, as in all post-hatchling archosaurs. The opisthotic extends laterally from the exoccipital to form the paroccipital process and the ventral margin of the postemporal fenestra. The paroccipital processes are dorsoventrally high, but short transversely. The ventrolateral border is much more steeply inclined and forms a notably ventrolaterally convex margin, which ventrally turns into the crista tuberalis.

Two foramina are present anteroventrally on either side of the occipital condyle. The small one piercing the exoccipital corresponds to a single opening for the passage of the cranial nerve XII. The larger opening corresponds to the metotic foramen for the joint exit of cranial nerves IX–XI, as in other pterosaurs (Wellnhofer, 1985; Bennett, 2001; Dalla Vecchia, 2009). Although in *Allkaruen* the foramen for cranial nerve XII is an independent opening from the metotic foramen, both foramina open within a shallow common oval recess, the paracondylar recess.

In the lateral view of the braincase, the columellar recess (probably enclosed between the prootic and the opisthotic, as is usual in reptiles) is visible as a large, drop-shaped opening separated dorsally from the anterior opening of the postemporal fenestra by the paroccipital process. The columellar recess is subdivided into a larger, anteromedially directed foramen and a smaller posterodorsally directed foramen. Whereas the former represents the foramen ovalis, the latter corresponds to a pneumatic foramen that communicates internally with a pneumatic cavity within the base of the paroccipital process (Fig. 1A, Fig. S1). In terms of its location this foramen appears to correspond to the pneumatic foramen of the posterior tympanic recess found in derived theropod dinosaurs (Witmer, 1997) and the allosauroid theropod *Sinraptor dongi* (Paulina-Carabajal & Currie, 2012). Although it is not possible to determine if the foramen for the cranial nerve V is completely enclosed by the prootic, this element forms at least the posterior margin of the opening.

The laterosphenoid is fused with the prootic posteriorly and with the frontal dorsally, following the postorbital process of the frontal. The postorbital process of the laterosphenoid is a narrow triangular projection adjacent to the posteroventral side of the postorbital process of the frontal. A narrow ventral projection of the laterosphenoid probably reaches the anteriormost part of the basisphenoid, forming the anterior margin of the foramen for the cranial nerve V.

The trigeminal foramen is large and ovoid, with a maximum diameter of 4 mm, and faces anterolaterally. There is no evidence of a separated foramen for the ophthalmic branch of the trigeminal nerve in the specimen, unlike the situation observed in *Anhanguera* (Witmer et al., 2003).

The orbitosphenoid and ethmoidal elements are missing; thus, the location and shape of cranial nerves I–IV remains unknown in *Allkaruen*. The symmetry of the anterior walls of the braincase enclosing the anterior section of the brain—frontal, laterosphenoids, and basisphenoid—suggests that the orbitosphenoid and ethmoid remained cartilaginous in this individual.

The elements of the basicranium, the basioccipital and basisphenoid, are firmly fused. The basioccipital forms the ventral portion of the occipital condyle and apparently the basal tubera. The occipital condyle is set off from the basioccipital body by a slightly constricted neck (Fig. S1). The lower part of the basioccipital, between the occipital condyle and the basal tubera, is strongly anteroventrally inclined, so that its ‘posterior’ surface faces rather ventrally and is set at an angle of approximately 100° with respect to the plane of exposure of the dorsal region of the occiput. Anteroventrally, the basioccipital strongly expands transversely towards the basal tubera, from a minimal width of 6.5 mm between the metotic foramina to approximately 12.5 mm across the basal tubera. The latter are anteroposteriorly elongated, but transversely narrow structures at the lateral extremes of the basioccipital expansion.

The ventral surface of the basisphenoid body is rhomboid in outline, with a tapering anterior end. Anteriorly, long and narrow basipterygoid processes extend for 11 mm anteroventrally from the basisphenoid body, from which they are offset dorsally by a distinct step. The processes are long and slender and diverge anteroventrally at an angle of approximately 35 degrees or slightly less (Fig. 1C). This contrasts with the morphology of most non-pterodactyloid pterosaurs, in which the processes are highly divergent (approximately 60°–70°), as in *Dorygnathus* (Padian, 2008), *Carniadactylus* (Dalla Vecchia, 2009), *Scaphognathus* (Wellnhofer, 1978), and *Cacibupteryx* (Gasparini, Fernández & De la Fuente, 2004), but is similar to the angle observed in *Rhamphorhynchus* (Wellnhofer, 1975). However, in *Allkaruen* the basipterygoid processes are separated over their entire length, and not connected by a bony web or plate, as seems to be the case in most, possibly all, pterodactyloids including the ornithocheiroids *Pteranodon* (Bennett, 2001), *Anhanguera* (Kellner & Tomida, 2000) and *Hongshanopterus* (Wang et al., 2008), the ctenochasmatines *Gnathosaurus* (Wellnhofer, 1970) and *Pterodaustro* (Codorniú, Paulina-Carabajal & Gianechini, 2015), the dsungaripteroid, *Dsungaripterus* (D Unwin, 2016, unpublished data), and the azhdarchoids *Caupedactylus* (Kellner, 2013) and *Tupuxuara* (D Unwin, 2016, unpublished data).

Two large pneumatic openings are present on the lateral side of the basisphenoid. The larger, more posteriorly placed opening is sited just posterior to the margin of the trigeminal foramen and corresponds to the anterior tympanic recess seen in many theropod dinosaurs (Witmer, 1997). A smaller, more anteriorly placed recess is separated from the anterior tympanic recess by a thin bony septum. This recess seems to be associated with the entrance of the carotid artery to the pituitary fossa and might be regarded as a basipterygoid recess. The pituitary fossa is developed as a large, anterodorsally opening depression on the dorsal side of the base of the basipterygoid processes.

The general morphology of the virtual cranial endocast is comparable to that described for the few other pterosaurs where it is preserved and can be studied. The endocast is bulbous, with short olfactory tract and bulbs, cerebral hemispheres with large and ventrally displaced optic lobes, and an extremely enlarged flocculus (Fig. 2). The left inner ear exhibits an anterior semicircular canal (ASC) that is considerably larger than the other two canals (Fig. 3). The dorsal region of the ASC is located ventral to the dorsal surface of the forebrain and approximately level with the olfactory tract.

Pneumatic cavities are present in the frontals, the ventral section of the exoccipital-opisthotic complex (Fig. 4), and in the form of the anterior and posterior tympanic recesses and basipterygoid recess described above. The cavities within the frontals are large and contrast with the camellate pneumatisation of some derived pterodactyloids (Kellner, 1996). The cavities that invade the basicranium are also large, equivalent to those observed in pterodactyloids such as *Pterodaustro* (Codorniú, Paulina-Carabajal & Gianechini, 2015). As noted above, the placement of the external foramina for these last recesses within the columellar recess indicate a tympanic origin for this pneumaticity. In turn, the pneumatic cavities that invade the exoccipital-opisthotic are smaller and affect internally the base of the paroccipital process and the neck of the occipital condyle on both sides of the braincase (Fig. 4).

The mandible is long (approximately 3.5 times the length of the preserved region of the skull), laterally compressed, and has concave alveolar and ventral margins in lateral view, so that the dentary is curved anterodorsally. Each dentary bears several separate anterior alveoli that occupy less than half of the preserved tooth row and a long and narrow alveolar groove posteriorly (Figs. 1D, 1E and Fig. S2), a morphology that is unique to *Allkaruen*. Although the symphysial region of the jaws has been lost due to recent erosion, the position of the two rami in the matrix indicate that the dentary symphysis was rather short.

The cervical centrum is approximately three times as long as wide, broader anteriorly than posteriorly, and lacks pre- and postexapophyses and hypapophysis. The prezygapophyses and postzygapophyses are connected by a thin lamina that is ventrally deflected along its anterior third, forming a short and triangular diapophyseal process that lacks an articular surface. Two pairs of pneumatic foramina pierce the vertebrae, one on the lateral surface of the centrum and the other on the ventral surface of the neural arch (Figs. 1F, 1G and S3). Both foramina are found at approximately the mid-length of the vertebra.

## Discussion

### Phylogeny

Phylogenetic analysis positions *Allkaruen* as the sister taxon of Monofenestrata, a clade that includes Wukongopteridae (*Darwinopterus* (Lü et al., 2010) and *Wukongopterus* (Wang et al., 2009) from the Middle-Late Jurassic of China) and more derived pterosaurs (Fig. 5). This location is supported by a mosaic of plesiomorphic and apomorphic character states to be found in *Allkaruen* (see SI).

There are two characters: the orientation of the occiput (#33); and the degree of separation of the basipterygoid processes (#37) where *Allkaruen* exhibits the plesiomorphic condition, while the derived condition is present in all mononfenstratans for which these characters can be scored. *Allkaruen* also exhibits the plesiomorphic condition for the length of the mandibular symphysis (#47) and development of postexapophyses (#71) although the plesiomorphic state is also found in at least one, or more, monofenstratans. There are four characters: angle of divergence of the basipterygoid processes (#34); elongation of the cervical centra (#73); height of the neural arch (#75); and height of the neural spines (#76) where all basal pterosaurs exhibit the plesiomorphic state, while *Allkaruen* shares the derived condition with monofenestratans, although for each of these characters the plesiomorphic condition is present in at least one monofenestratan (see SI). There are a further six characters (#12, #32, #36, #53, #61, #62) for which *Allkaruen* exhibits a derived state, that is found in monofenestratans and at least one, or more (relatively derived), basal pterosaurs.

### *Allkaruen* and the evolution of the pterosaur neurocranium

The intermediate phylogenetic position of *Allkaruen* and the exceptional three-dimensional preservation of the braincase provide new insights into the transformation of the neurocranium, typical of basal pterosaurs, into the highly derived condition present in pterodactyloids (Witmer et al., 2003) (Figs. 6C–6H). Descriptions of the neurocranial anatomy of pterosaurs have so far been limited to two basal forms (Newton, 1888; Edinger, 1927; Wellnhofer, 1975; Witmer et al., 2003) and a few pterodactyloids (Edinger, 1927; Edinger, 1941; Kellner, 1996; Lü et al., 1997; Bennett, 2001; Witmer et al., 2003; Eck, Elgin & Frey, 2011). Detailed accounts of endocranial morphology (brain and inner ear) have been published for only two pterosaurs, the non-monofenestratan breviquartossan *Rhamphorhynchus* and the derived pterodactyloid *Anhanguera* (Witmer et al., 2003).

Comparisons with the virtual endocasts of *Rhamphorhynchus* and *Anhanguera* (Fig. 6), show that *Allkaruen* shares plesiomorphies and apomorphies with one, or the other, of these two taxa and also exhibits features with a morphology that can be interpreted as intermediate between that of *Rhamphorhynchus* and *Anhanguera*. A seemingly plesiomorphic feature of the brain and inner ear of *Allkaruen* is the subhorizontal orientation of the frontal when the long axis of the LSC is oriented horizontally (Fig. 6B). This is comparable to the condition in *Rhamphorhynchus* (Witmer et al., 2003), but contrasts with the ventrally deflected frontal of *Anhanguera* (Witmer et al., 2003). Note that, here, we use the orientation of the LSC purely as a descriptive term, and do not infer any particular orientation of the head in the taxa under consideration (Marugán-Lobón, Chiappe & Farke, 2013).

Four features of the braincase and endocranium of *Allkaruen* show an intermediate condition between non-monofenestratan breviquartossans and pterodactyloids.

(I) The dorsalmost point of the ASC is located approximately at the same level as the dorsal margin of the forebrain in *Rhamphorhynchus* (Witmer et al., 2003) (Fig. 6F) and *Parapsicephalus* (Newton, 1888). By contrast, it is located at the midheight of the forebrain in *Allkaruen* (Fig. 6G), and ventral to the olfactory tracts in several pterodactyloids including *Pteranodon*, *Tapejara*, and *Anhanguera* (Witmer et al., 2003) (Fig. 6H).

(II) The orientation of the occiput and occipital condyle is intermediate between that of non-monofenestratan breviquartossans, in which the occiput is mainly vertical, with a posteriorly oriented occipital condyle, and pterodactyloids, in which the occiput faces posteroventrally, or even ventrally. In *Allkaruen* the dorsal section of the occiput faces posterodorsally, whereas the occipital condyle is inclined posteroventrally an intermediate condition also found in *Darwinopterus* (D Unwin, 2016, unpublished data).

(III) In dorsal view, the lateral margin of the flocculus in *Rhamphorhynchus* does not extend as far laterally as the lateral margin of the cerebral hemisphere, whereas these two are about level in *Allkaruen*. By contrast, in *Anhanguera* the flocculus extends well beyond the lateral margin of the cerebral hemisphere.

(IV) The ratio between the complete length of the brain and the height of the hindbrain, when seen in lateral view, is 0.44 in *Rhamphorhynchus* and 0.69 in Anhanguera, whereas this ratio is 0.6 in Allkaruen.

While the endocast morphology of *Allkaruen* seems, in many respects, to be intermediate between that of basal pterosaurs and pterodactyloids, it also demonstrates an important derived feature that is shared with monofenestratans. In basal taxa such as *Parapsicephalus* (Newton, 1888) and *Rhamphorhynchus* (Witmer et al., 2003) the optic lobes lie at the same level as the forebrain (Fig. 6F). *Allkaruen*, *Darwinopterus* (D Unwin, 2016, unpublished data) and pterodactyloids (Edinger, 1941; Bennett, 2001; Witmer et al., 2003; Eck, Elgin & Frey, 2011) show a derived state, pronounced flexure of the brain that displaces the optic lobes ventrally (Figs. 6G and 6H), a condition convergently present in birds (Walsh & Milner, 2011). This arrangement represents a major reorganization of neurocranial architecture and while its significance has yet to be established, it seems to be an important innovation in pterosaur evolution.

Finally, *Allkaruen* exhibits a neuroanatomical feature that is more derived than in other pterosaurs. The ASC is slightly larger than the other two semicircular canals in *Rhamphorhynchus* (25%) and *Anhanguera* (30%), but much larger (40–50%) in *Allkaruen*.

### Pterosaur evolution

The discovery of strongly correlated character state distributions in *Darwinopterus* led Lü et al. (2010) to suggest that major anatomical regions might have behaved as integrated modules that changed at different times and rates during pterosaur evolution. However, *Allkaruen* demonstrates that, whereas modular evolution might have operated at an inclusive morphological level (e.g., skull + neck versus the remainder of the postcranium), evolution within at least one of these modules (the neurocranium and braincase) seems to have followed a mosaic pattern.

The late Early-early Middle Jurassic age of *Allkaruen* (Cúneo et al., 2013) also provides new information on the timing of transformations during the evolution of the derived pterodactyloid skull from that of basal pterosaurs. The derived features of the cranium of *Allkaruen* indicate that some typical “pterodactyloid” skull features had already evolved by the time of the Early/Middle Jurassic boundary (Fig. 7), before the origin of pterodactyloids and the appearance of their modified postcranial skeleton. Prior to this discovery, a large suite of cranial features was presumed to have appeared somewhat later, during the late Middle to Late Jurassic, the age of the basal monofenestratans, *Darwinopterus* (Lü et al., 2010) and *Wukongopterus* (Wang et al., 2009) and the oldest pterodactyloids (Andres, Clark & Xu, 2014).

Unfortunately, the Early-Middle Jurassic is a period with a very poor pterosaur fossil record, in contrast to the relatively diverse assemblage of pterosaurs known from both the Late Triassic and the Late Jurassic–Cretaceous (Barrett et al., 2008; Butler, Benson & Barrett, 2013; Benson et al., 2014). The early evolutionary origin and diversification inferred for derived pterosaurs (Fig. 4), adds further evidence in support of the hypothesis that the origin and diversification of major vertebrate lineages (e.g., dinosaurs (Allain & Läng, 2009; Pol & Rauhut, 2012), crocodyliforms (Pol & Gasparini, 2009), turtles (Sterli, Pol & Laurin, 2013), mammals (Luo et al., 2011)) occurred prior to the Early/Middle Jurassic boundary (Allain & Läng, 2009; Cúneo et al., 2013). This pattern was previously obscured by the worldwide poor fossil record of terrestrial vertebrates during this evolutionarily critical period of time.

##  Supplemental Information

10.7717/peerj.2311/supp-1 Supplemental Information 1 Supplementary Material

10.7717/peerj.2311/supp-2 Data S1

10.7717/peerj.2311/supp-3 Data S2

## Additional Information and Declarations

### Data Availability

The following information was supplied regarding data availability: The research in this article did not generate any raw data.

### New Species Registration

The following information was supplied regarding the registration of a newly described species: Publication LSID: urn:lsid:zoobank.org:pub:48910653-0343-4A8D-911F-3498A755F305. New taxon LSID: urn:lsid:zoobank.org:act:C545BD35-B448-4D47-A2A6-14215E9E3155.
