# Supplementary material for: A Jurassic pterosaur from Patagonia and the origin of the pterodactyloid neurocranium
Source: PeerJ. 2016 Aug 30;4:e2311. doi: 10.7717/peerj.2311 (PMC5012331; doi:10.7717/peerj.2311)
Supplement: Supplemental Information 1 [file peerj-04-2311-s001.doc]

Supplementary Materials

**A Jurassic pterosaur from Patagonia and the origin of the pterodactyloid neurocranium**

Laura Codorniú^1^, Ariana Paulina-Carabajal^2^, Diego Pol^3^, David Unwin^4^ & Oliver W. M. Rauhut^5^

^1^CONICET, UNSL. ^2^CONICET, Instituto de Investigaciones en Biodiversidad y Medioambiente (INIBIOMA), Quintral 1250, 8400 San Carlos de Bariloche, Argentina. ^3^CONICET, Museo Paleontológico Egidio Feruglio, Av. Fontana 140, 9100 Trelew, Argentina. ^4^UNWIN, School of Museum Studies University of Leicester, 19 University Road, Leicester LE1 7LF, United Kingdom. ^5^SNSB, Bayerische Staatssammlung für Paläontologie und Geologie, Department of Earth and Environmental Sciences and GeoBioCenter, Ludwig-Maximilians-University, Richard-Wagner-Str. 10, 80333 Munich, Germany

**TABLE OF CONTENTS:**

| **1 Additional ANATOMICAL FIGURES** | **3** |
| --- | --- |
| **2. PHYLOGENETIC ANALYSIS** | **6** |
| **2.1 Support measures** | **6** |
| **2.2 Character list** | **8** |
| **2.3 Data matrix** | **13** |
| **2.4 List of synapomorphies** | **23** |
| **3. REFERENCES** | **30** |

**1. ADDITIONAL ANATOMICAL FIGURES**


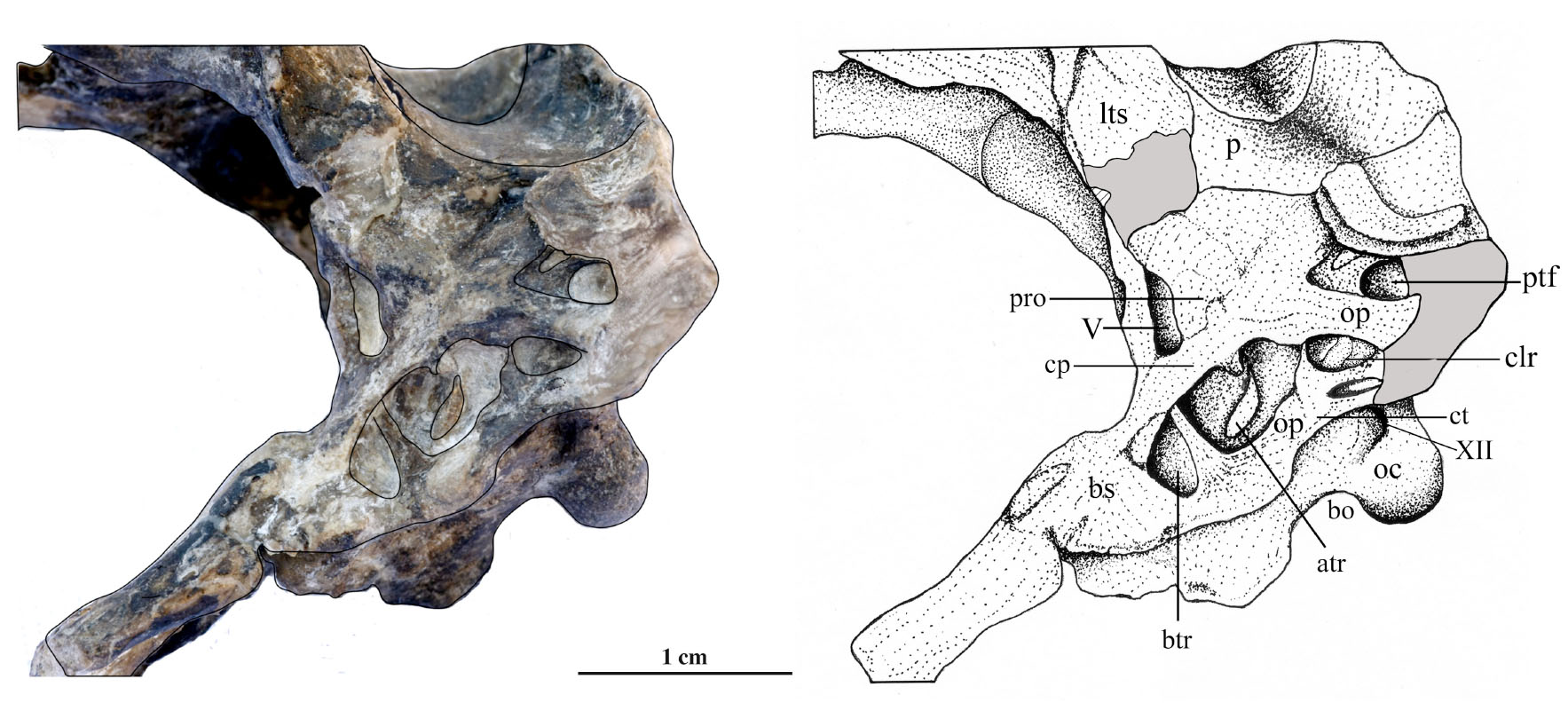


**Suppl-fig. 1:** Details of the left ventrolateral view of the skull of *Allkaruen koi* gen. et sp. nov. MPEF-PV 3613. Abbreviations: atr, anterior tympanic recess; bo, basioccipital; bs, basisphenoid; btr, basipterygoid recess; clr, columellar recess; cp, crista prootica; ct, crista tuberalis; lts, laterosphenoid; op, opisthotic; p, parietal; pro, prootic; oc, occipital condyle; ptf, postemporal fenestra; V, XII, cranial nerve foramina.

**
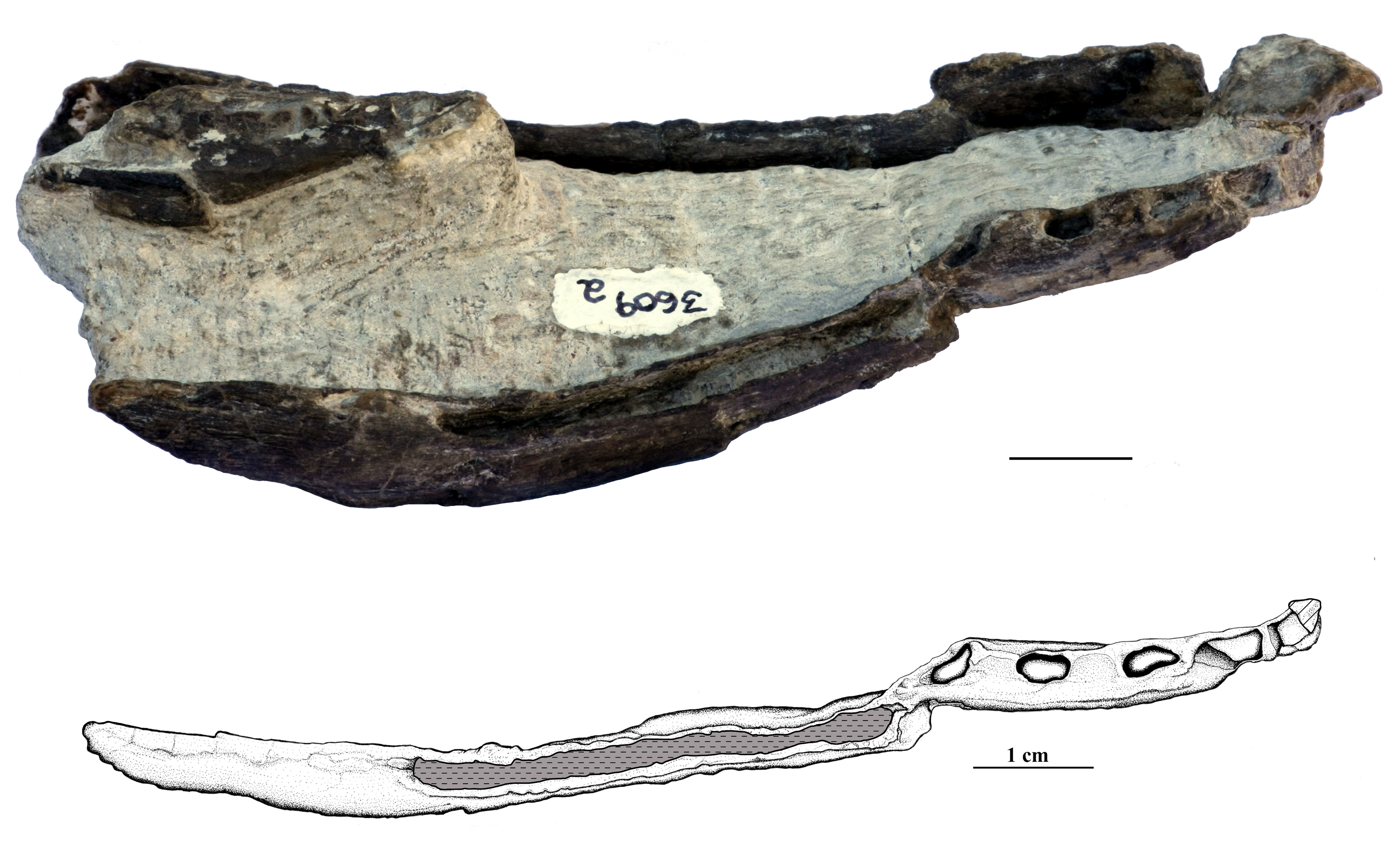
**

**
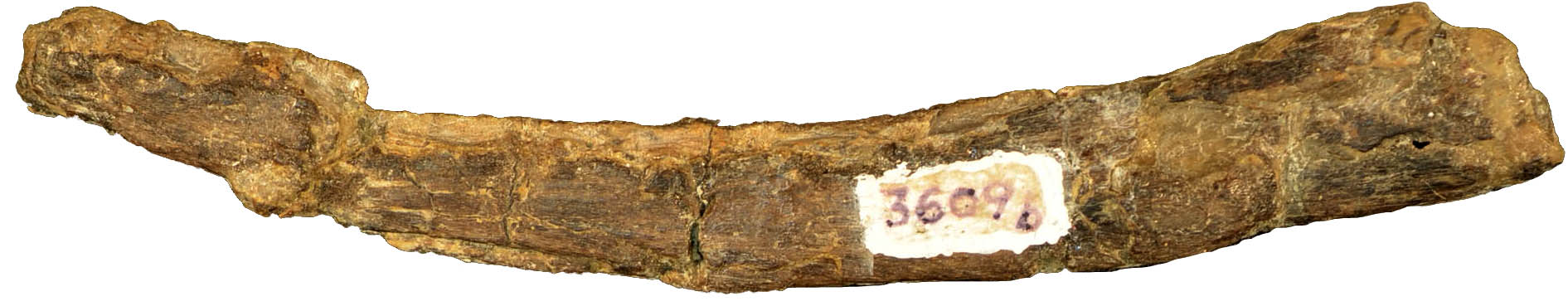
**

**Suppl-fig. 2:** Mandible of *Allkaruen koi* gen. et sp. nov. MPEF-PV 3609 in dorsal (photo and drawing) and left lateral views.

**
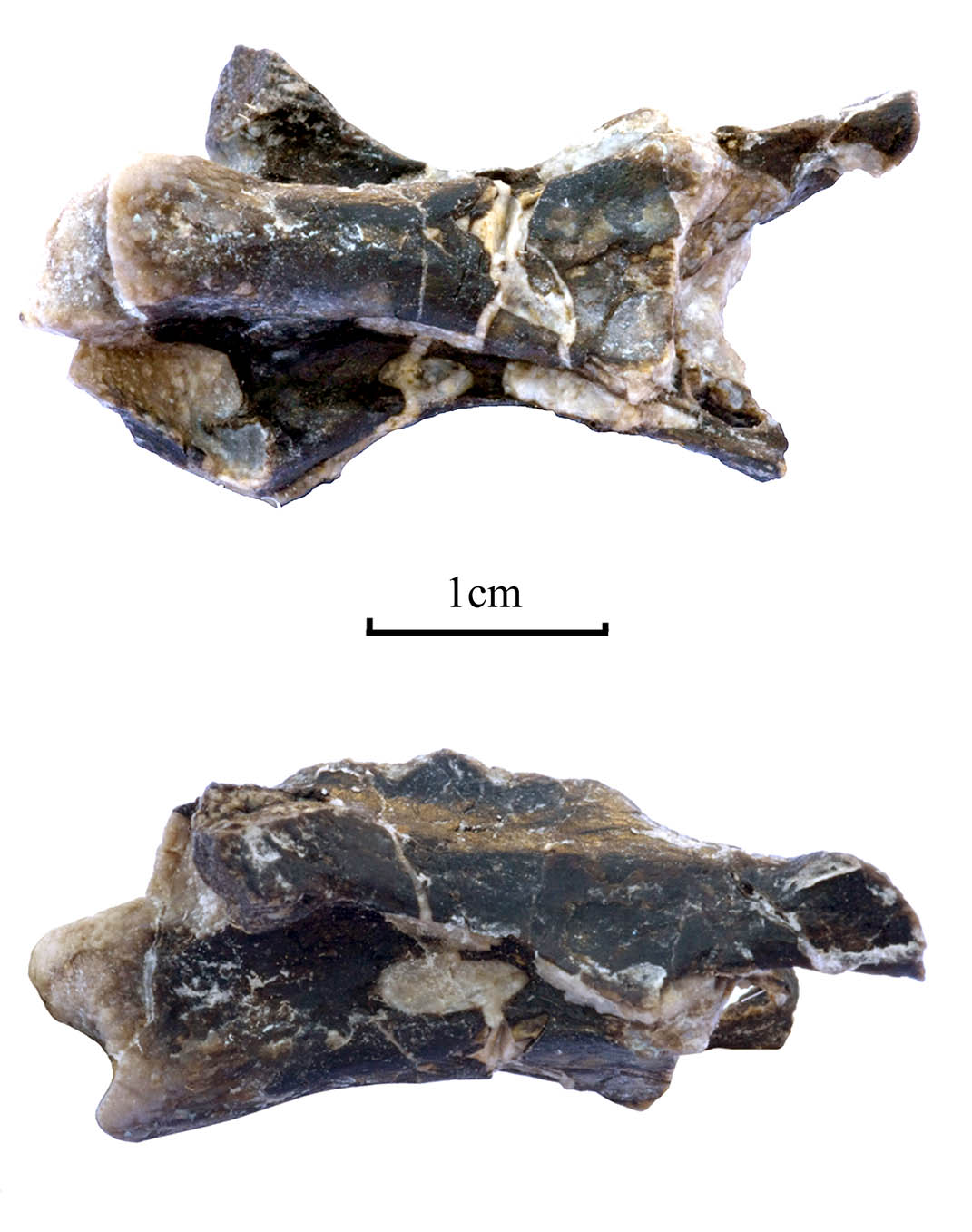
**

**Suppl-fig. 3:** Cervical vertebra of *Allkaruen koi* gen. et sp. nov. MPEF-PV 3615 in ventral and right lateral views.

**2. PHYLOGENETIC ANALYSIS**

**2.1 Support measures:** Bootstrap frequencies and Bremer support values were calculated using TNT to evaluate the robustness of the nodes of the most parsimonious trees. Bremer support (Bremer, 1994) was used through the script provided by TNT v.1.1 that utilises a combination of heuristic searches saving suboptimal trees and negative constraints for monophyly. One thousand bootstrap pseudoreplicates were generated using a heuristic tree search of 10 Wagner trees (with random addition sequences) followed by TBR. Results of these replicates were summarized using absolute frequencies for each group that was present in the strict consensus.

The following tree (reproducing Figure 5) includes the Bremer support value and Bootstrap frequencies for each of the nodes present in the strict consensus tree. Support values were derived from the analysis based on both the type and the referred specimens of *Allkaruen*. Calculation of support values from the analysis based on only the holotype yielded virtually identical results. The only exceptions were in the nodes Monofenestrata and Pterodactyloidea, for which support values of the latter analysis are given in parentheses below those of the analysis based on both the type and the referred specimens.


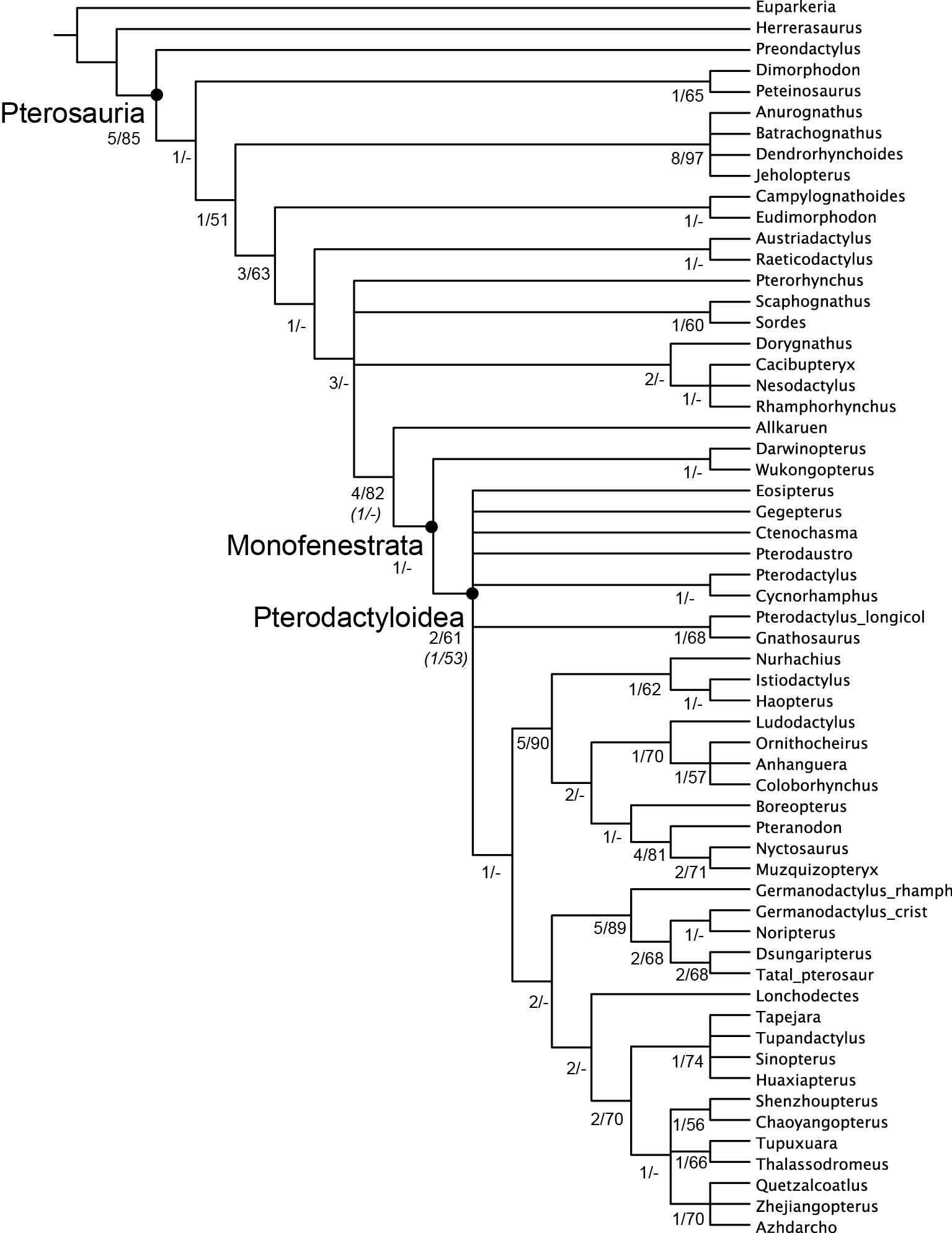


**2.2 Character list:** Character and character state definitions are listed below for the data matrix used in the phylogenetic analysis. Characters were taken from the study of Lü et al. (2010). All modified characters and new characters are indicated in parentheses after the definition. The multistate character 33 was treated as ordered.

1. Tip of rostrum downturned: absent (0); present (1).
2. Tip of rostrum laterally expanded: absent (0); present (1).
3. Tip of rostrum: laterally compressed (0); dorsoventrally compressed (1).
4. Rostrum: high with convex outline (0); low with straight or concave dorsal outline (1); anterior region of rostrum low, but antorbital region expanded dorsally (2).
5. Prenarial rostrum: less than (0); or more than 50% skull length (1).
6. Rostral index (antorbital length divided by maximal skull height [excluding cranial crests]): 1.5 or less (0); 1.5-3.0 (1), >3.0 (2). (modified from Lü et al. 2010)
7. Preorbital rostrum: <80% skull length (0); >80% (1).
8. Dorsal margin of nasoantorbital opening bounded by slender bar: absent (0); present (1).
9. Premaxillary crest: absent (0); low, rounded, confined to rostrum (1); comb-like free margin and extends from above anterior end of nasoantorbital fenestra to apex of skull (2); extends from tip of rostrum to apex of skull and confluent with fronto-parietal crest (3); tall, narrow, stands on anterior half of premaxillae (4); keel-like, anterior margin extends no further forward than midpoint of nasoantorbital fenestra (5).
10. Skull broad with very short preorbital region: absent (0); present (1).
11. Ventral margin of skull: straight (0); downcurved caudally (1).
12. Posterior extent of premaxillae: terminates level with frontals (0); overlaps frontals (1).
13. Nasal process of maxilla: vertical-subvertical (0); inclined backwards (1); absent (2).
14. Maxilla-nasal contact: narrow (0); broad (1); lost (2).
15. Antorbital fossa on the ascending process of the maxilla: present (0), absent (1) (new character)
16. Nasal opening: terminal (0); subterminal (1).
17. External nasal opening: height similar to or greater than anteroposterior length (0); elongate (1).
18. Nares: form the largest skull opening (0); smaller than the orbit or nasoantorbital opening (1).
19. Dorsal border of the antorbital fenestra lies below the mid-height of the naris: absent (0); present (1). (modified from Lü et al. 2010)
20. Antorbital fenestra: less than twice as long as it is deep (0); at least as twice as long as it is deep (1).
21. Naris and antorbital opening: separate (0); confluent (1).
22. Nasoantorbital fenestra: <40% skull length from tip of rostrum to posterior margin of orbit (0); >40% (1).
23. Posterior margin of antorbital or nasoantorbital fenestra: straight (0); concave (1).
24. Orbit shape: subcircular (0); tall, oval (1).
25. Orbit: smaller than or similar in size to antorbital opening (0); larger (1).
26. Dorsal margin of orbit: above dorsal margin of antorbital or nasoantorbital opening (0), level with dorsal margin of nasoantorbital opening (1); or below it (2). (modified from Lü et al. 2010)
27. Frontal extends anterior to the lacrimal-jugal bar: absent (0); present (1).
28. Fronto-parietal crest: absent (0); flange-like, short (1); flange-like, elongate (2); rod-like, short (3); rod-like, elongate (4); sail-like (5).
29. Posterior region of skull rounded: absent (0); present (1).
30. Squamosal position: above, or level with the orbit (0); entirely below the orbit (1).
31. Supratemporal fenestra largest skull opening after the orbit: absent (0); present (1).
32. Separation of supratemporal fenestrae: wide, formed by a dorsally facing bridge of the parietals (0); narrow, parietals form sharp saggital crest (1) (new character)
33. Occiput: faces posteriorly (0) or posteroventrally (1); or ventrally (2). (ordered, modified from Lü et al. 2010)
34. Occipital condyle: on the posterior (0) or posteroventral (1) face of the occiput (new character)
35. Distal ends of paroccipital processes: unexpanded (0); rounded, tongue-like flange (1).
36. Basipterygoid processes: widely diverging (angle between processes ≥35°) (0); narrow (angle < 35°) (1) (new character)
37. Basipterygoid processes: separate over their entire length (0); connected by a bony web, only the distal articular ends might be separate (1) (new character)
38. Quadrate: vertical (0); inclined backward (1); subhorizontal (2).
39. Palatal elements reduced to thin bars of bone: absent (0); present (1).
40. Position of the jaw joint: under the posterior third of the orbit (0); under the middle third of the orbit (1); under the anterior third of the orbit (2).
41. Palatal ridge: absent (0); present (1).
42. Dentary: <50% length of lower jaw (0); >50% (1).
43. Anterior tip of the mandible: horizontal (0); downturned (1).
44. Anterior end of the lower jaw: unexpanded (0); expanded transversely (1). (modified from Lü et al. 2010).
45. Anterior end of mandibular symphysis: laterally compressed or as wide as deep (0); dorsoventrally compressed (1).
46. Mandibular symphysis: unfused (0), fused (1) (modified from Lü et al. 2010).
47. Length of symphysis: less (0) or more (1) than 30% of the length of the mandible. (Andres and Ji 2008)
48. Mandible tips fused into a short symphysis bearing a forward projecting 'tooth-like' prow and a number of large, fang-like, procumbent teeth forming a fish grab: absent (0) present (1).
49. Anterior end of dentary: level (0); dorsally expanded forming low rounded eminence (1); high rounded eminence (2).
50. Mandibular rami; level with symphysis (0); elevated well above level of symphysis (1).
51. Dorsal margin of the dentary: more or less straight (0); markedly concave (1); convex (2); sinuous (3) (new character).
52. Dentary bony sagittal crest: absent (0); present (1).
53. Pre-dentary region relatively deep with convex dorsal profile, dentary tapers anteriorly (0); predentary region and dentary of similar depth, dentary of similar depth for much of its length (1).
54. Dimorphodontid dentition: absent (0); present (1).
55. Teeth are small, peg-like and widely spaced: absent (0); present (1).
56. Multicusped teeth: absent (0); present (1).
57. Rostral dentition: more than 11 pairs of teeth (0); less (1).
58. Rostral dentition: more than nine (0); nine or less relatively straight (or slightly recurved teeth (1).
59. Mandibular dentition: more than six pairs of teeth (0); six or less (1).
60. Heterodonty in the mandibular dentition: present (0); absent (1).
61. Anterior dentary teeth: distance between teeth less (0) or more (1) than mesiodistal diameter of the teeth. (new character)
62. Posterior dentary teeth: distance between teeth less (0) or more (1) than mesiodistal diameter of the teeth. (new character)
63. Dentition: present (0); absent (1).
64. Largest teeth in caudal half of dentition: absent (0); present (1).
65. First three pairs of teeth large, 4th-6th small, 7th-9th large: absent (0); present (1).
66. Short, broad teeth in at least part of the dentition: absent (0); present (1).
67. Dentition: extends to jaw tips (0); jaw tips toothless, but followed by tooth row (1).
68. Teeth relatively elongate with a long cylindrical crown of even width and a short tapering distal tip: absent (0); present (1).
69. Total of more than 192 long, fine teeth: absent (0); present (1).
70. Laterally compressed, triangular teeth in at least part of the dentition: absent (0); present (1).
71. Postexapophyses on cervical vertebrae: absent (0); present (1).
72. Lateral pneumatic foramen on centrum of the cervical: absent (0); present (1).
73. Mid-series cervicals: short (length/mid width ≤ 2.5) (0); elongate (2.5-5) (1); very elongate (>5) (2). (modified according to Andres and Ji 2008)
74. Cervical ribs: present (0); highly reduced or absent (1).
75. Neural arch of cervicals: high (0); depressed down onto, or even confluent with, the centrum (1).
76. Neural spines of mid-series cervicals: tall, blade-like (0); tall, spike-like (1); low or absent (2).
77. Notarium: absent (0); present (1).
78. Number of caudal vertebrae: more than 15 (0); 15 or fewer (1).
79. Combined length of caudal vertebrae: longer than the dorsal series (0); shorter (1).
80. Filiform extensions of zygapophyses and hypapophyses: absent (0); present (1).
81. Sternum: absent (0); rectangular (1); triangular (2); semicircular (3), square with posterolateral projections (4).
82. Cristospine of sternum: unconstricted (0); constricted (1).
83. Coracoid: less than two thirds length of scapula (0); from at least two thirds up to similar length to scapula (1); longer than scapula (2).
84. Coracoid with well-developed brachial flange: absent (0); present (1).
85. Coracoidal contact surface with sternum: articulation surface flattened, lacking posterior expansion (0); articulation surface oval, with posterior expansion (1).
86. Shape of scapula: elongate (0); stout with constricted shaft (1).
87. Appendicular bones with thin cortex and wide lumen: absent (0); present (1).
88. Forelimb: up to 2.5 times length of hind limb (f+t+mt) (0); 2.5-3 times length of hind limb (1); 3-4 times length of hind limb (2); at least 4 times length hind limb (3).
89. Pneumatic opening in palmar surface of humerus: absent (0); present (1).
90. Pneumatic opening in anconal surface of humerus: absent (0); present (1)
91. Deltopectoral crest of humerus: small (0); large and subtriangular with apex directed proximally (1); proximo-distally elongate, rectangular (2); hatchet-shaped (3); tongue-shaped with necked base (4); antero-posteriorly elongate with rectangular shape (5); antero-posteriorly elongate, main axis directed anteromedially, terminal margin rounded (6); warped (7); distally expanded (8).
92. Distal end of humerus: D-shaped (0); triangular (1).
93. Diameter of radius: more than half the ulna (0); less (1).
94. Ulna: considerably shorter than dorsals+sacrals (0); similar in length to dorsals+sacrals (1).
95. Ulna: less than 133% humerus (0); 133-150% (1); >150% (2).
96. Ulna/tibia ratio: 0.9-1.2 (0), 1.2-1.4 (1); >1.4 (2); <0.9 (3).
97. Ornithocheiroid carpus: absent (0); present (1).
98. Pteroid: less than 30% length humerus: (0); 30-60% (1); >60% (2).
99. Metacarpals I-III: disparate lengths (0); the same length (1).
100. Contact between distal syncarpal and metacarpals I-IV: all four in contact (0); only I and IV contact syncarpal (1); only IV contacts the syncarpal (2).
101. Wing-metacarpal : humerus ratio: less than 0.8 (0); between 0.8 and 2.2 (1); > 2.2 (2).
102. Unguals of manus and pes: similar in size (0); manual unguals twice the size, or more, of pedal unguals (1).
103. Manus digit iv (wing-finger): 57.5%, or less, of total forelimb length (0): >57.5% (1); >65% (2).
104. Proceeding distally, wing phalanges 1-4 exhibit a rapid decline in length, contributing 40%, 30%, 20% and 10% to the wing-finger respectively: absent (0); present (1).
105. Manus digit iv (wing-finger) phalange 1 compared to length of tibiotarsus: shorter (0); 1 to 1.5 times longer (1); 1.5-2.0 times longer (2); more than twice the length (3).
106. Longitudinal ventral ridge on wing-phalanges two and three: absent (0); present (1).
107. Wing-finger phalanges with deep posterior groove: absent (0); present (1).
108. Contribution of wing-phalange 1 to wing-finger length: less than 30% (0); 30-40% (1); more than 40% (2).
109. Manus digit iv (wing-finger) phalanges: decline in length distally (0); phalanges 2 and/or 3 longer than phalange 1 (1).
110. Preacetabular process of ilium: similar length to postacetabular process (0); longer (1).
111. Anterior profile, in lateral view, of pubis: convex or straight (0); slightly concave (1); deeply concave (2).
112. Pubis and ischium: unfused (0); fused to form a plate with a straight ventral margin that meets the posterodorsal margin at an acute angle (1); with convex ventral border, ischium that projects below level of the pubis and obtuse posterior apex (2); dsungaripterid ischiopubis (3).
113. Prepubis: distal expansion longer than broad or similar width to length (0); transversely expanded (1); cojoined prepubes forming H shape (2).
114. Leg length (Femur+Tibia): less than 1.5 x length of dorsal+sacral vertebral series (0); more than 1.5 times (1).
115. Femur caput: directed inward at about 135° (0); directed steeply almost parallel to long axis of femur shaft (1).
116. Strongly bowed femur: absent (0); present (1).
117. Prominent anteriorally directed tubercle on dorsal apex of external trochanter of femur: absent (0); present (1).
118. Pneumatic opening in posterior face of collum femoris: absent (0), present (1).
119. Fibula: subequal in length (0), or less than 80 percent the length of the tibia (1); reduced to a small splint or lost altogether (2).
120. Length of metatarsal III compared to tibia: <30% (0); >30% (1).
121. Length of metatarsal 4: similar in length of metatarsals i-iii (0); shorter than metatarsals i-iii (1).
122. Fifth pedal digit: two phalanges or more (0); one very short phalange, or less (1).
123. Phalange two of pedal digit v with distinctive angular flexure at mid-length, such that the distal half of the phalanx lies at 40-45° to the proximal half: absent (0); present (1).

**2.3 Data matrix:** The following data matrix details the character scorings used in the phylogenetic analysis. Character scorings enclosed between braces or parentheses indicates polymorphic scorings and dashes represent inapplicable data.

Euparkeria

0000000-0000010001010-101010000000000000?1000000000000000000000000000000000000000-000100000000000-0-0????????000-01100?1000

Herrerasaurus

0000010-0000110001000-10100000000000000001000000000000000000000000000000100000000-000000000000000-0-000-0-0--010-001000101-

Preondactylus

0000010-00?011?101000-01100000????0????0?00000000000010100000000000000??000??000??00?010??1?00000000101000001001?0000?1100?

Dimorphodon

0000010-0000100100000-010100000?0?0??000?100000000[0 3]001000000100000000001000?0001??100011001000[0 1]0000011100000100100000000000

Peteinosaurus

?????????00??????????????????????????????1?000000000010000001000000000??0?0?0001?0100011?010000000001110000010?10000??01000

Anurognathus

00?0000-010000?000000-?01000000???????10?10000000000?010110111000000000?010?0010??10?01100200111000001101002010??0000011000

Batrachognathus

00?0000-010000?000000-?00?0000????0???10?100000000?010100001??00000000??0???0010101?001??0200??1??0??1???????1?????00?11000

Dendrorhynchoides

00?000?-01??00?00??0?-?????00?????????1????00?0000?0?010??????000000000?010?0010??100012?0200101000001102?0201???0100?11000

Jeholopterus

00?0000-01??0??0????0-????000??????????0???00?0000?0?010??????00000000??01??0010??100012?020011200?00110200101??00000?11000

Austriadactylus

0001010-20?111?111000-?0100000???????0?0?1?00????00?000100001000000000??00000000?????01????00???0????1?0?00?1??????0??1????

Campylognathoides

0001010-0001111111000-001000001???000100?11000001000000000001100000000010000000140100013003000010[0 1]0011202000111210000001100

Eudimorphodon

0001010-0001110111000-001000001?0?0??100?110000010000001000010000000000?000?000040110012003000[0 1][0 1]010011?0?000101210[0 1]00011100

Raeticodactylus

0001000-201?110111000-0110?0000???0??100?1000?002031000100000000000000010?000?????????1?0?30??01??0????0100?1?????00001????

Scaphognathus

0001010-1011110111000-00100000010?000100010001000000100011111100000000??000000012010?01200400022011011101000110210000?11101

Sordes

0001010-001111?111000-001000000???0??100?10001000000100011111100000000??000000012?1000130040002101101110100011021000001[0 1]101

Pterorhynchus

0001010-201111?1110?0-001?00000???0??1?0?1000??0000000001101??00000000??0?00?001??????12???00022?1?01?1010001????0?0?????0?

Cacibupteryx

00010?0-0011111111010-1110000000000000000????????????00?1?????0?????0??????????????????????????????????????????????????????

Dorygnathus

0001010-0011111111100-001000000?0?000100?10001010020100010011100000000??000000012010001200400022010011101000110210000011100

Nesodactylus

????????????????????0-??????????????????????????????????????????????????0000???12010001?00400?2?0?101?20?01001020?0????????

Rhamphorhynchus

0001020-0011111111110-1010000000000101000100011100201000100111000000000100000001?010001300400022011011203010010210000011100

Darwinopterus

00010210201122-110001111[0 1]100000111010101110001000000100000011100000000111012000130110012006000?00?0010100000112210000011101

Wukongopterus

000?02???0?????11????????????????????????10001?0000010000001??0?000000?010120001??11?0?2?0??00200?001010000011???0000011101

Pterodactylus

00010200001122-1100010001100110?2?0?0201010001100000100000011100000000001112011020100012005000[0 1]002102110[0 1]00[0 1]01121000001111?

Cycnorhamphus

00110200001122-1??0?1000?100110?2?0??2?201001110000010001101110000000000010001103011001200500013021020101001011210000?101??

Eosipterus

????????????????????????????????????????????????????????????????????????11120110??1100110050000001102010100001???000001111?

‘Pterodactylus’_longicol

01111200001122-1??0?10?0?1001?0?2?0??2010101111000001000000111000001001?11120110??110010005000030?1120001001011211000?1011?

Gnathosaurus

01111210201122-11?0010001100110121011201010111100000100000011100000100?????????????????????????????????????????????????????

Gegepterus

00111210001122-11?0010001000110?2?0??201?10?11?0?00010000001?1000001101111120??????10?1?????????0??????0100?01?????0??11???

Ctenochasma

00111210[0 2]01122-1??001000?000110?2?0??201?10011100000100000011100000110??11120110?01?0011?05000[0 1]0?1102010100101?21000001111?

Pterodaustro

00111210001122-1??001000?100110?2?0?1201?10011100010100000011000000110111112?0103011001000500?000?112010100101121000001111?

Istiodactylus

00110100001122-1??-01101?[0 1]00000???0??102010011000000100000010?000000011101011????020011301711?211?012?1020010?????10002????

Nurhachius

0011021000?122-110-0110111?0??0??????10201001110000010000001110?000001?101011?????20011?0?7?1?2???112?10200201????100?201??

Haopterus

001101?000??22-1??-010?????0?????????10??10011100000100000011100100001??010?0???3020?11???711?1?11002110?0010???????????11?

Ornithocheirus

01010210101122-1??-?1011?001000?1?0?1102110101100001100000011100100000?????????????????????????????????????????????????????

Anhanguera

01010210101122-110-01011100000011101110211010110000[0 1]100000011100100000110101111031201113017111?11211211020010101?1100020???

Coloborhynchus

01010210101122-1??-?1011?000000?11011102110101100001100000011100100000110101111031201113017111111?11211020010101?010002011?

Ludodactylus

01010210001122-110-0101?1001000???0??102?10?01100000100000011100100000?????????????????????????????????????????????????????

Boreopterus

00011210[0 1]01122-1??-01011??00000???0??102?100011000001000000111001000001?0101?110??????11????1111?111211020010????0100?2011?

Nyctosaurus

00011210001122-1??-?1010?002000???0111020100011001001---111---1-------100101111010210112108?112212?23?[0 1]0300201112010002111?

Muzquizopteryx

???0???0001122-1??-?1?10??02000???0??1?2?1???????1?01---111-??1-------??010?111011?1?11??08?11?????2?????????1??2010002??1?

Pteranodon

00011210001122-1??-?1011?00200011?0111020100011001101---111---1-------1101011110112001111071111011123110300101012010001011?

Germanodactylus_crist

00010200200122-1100010001100000?1?1??1?1?10001100000100000001101011000?101000?10??1?0001?05001130?102010[0 1]0010????101101111?

Germanodactylus_rhamph

00010200200122-1100011001100000?1?1??101?10001100000100000001101010000?101000110??100011?05001200??020000001012?11011?1011?

Dsungaripterus

00010200200122-1??-?1000?00300011?1?110101000110001000000000-00101100011011011101011000?00500???0???20?0?00??123?101101????

Tatal_pterosaur

00010200200122-1??-?1001??03000???1??101?10001?000?000000000??010110001101100?????11?00200500?130?1120?0100?0????1011010???

Noripterus

?????????0???????????????????????????????1?0?1???????00???00110??1100?11010???????????0?00500?030?1120?0100?01????01101111?

Lonchodectes

000?????[0 1]0?????1????????????????????????110011?000?[0 1]100000001100000000101112???????1??0?1050??????????????????????0011?????

Tapejara

10020100401122-1??-?1101?2130001100??1020100011000211---111---1-------1011120???1011001?00500?1?0??120?1?00?0102??000010?1?

Tupandactylus

10020210401122-1??-?1101?214000?1?0??1?2?10001?000?11---111---1-------?????????????????????????????????????????????????????

Sinopterus

1002020000?122-1??-?1101?213000???0??101?100011000[0 2]11---111---1-------1?111201101011?011??5?0113021?20011002010211001?1011?

Huaxiapterus

1002020040?122-1??-?1100?214000???0??1010100011000211---111---1-------??1112?110??110?111050011302112001100?0???11001?10???

Shenzhoupterus

00020211001122-1??-?1101?215000???0??1?2?10001?001?01---111---1-------??11120?10??11?011??5001230?12200110020????100???0?1?

Chaoyangopterus

0002?2?100??22-1????1????????????????1???100011001?0?---111---1-------??1112??1???1???11??????13?2122?0100020????100??10?1?

Tupuxuara

00020210301122-1??-?1101?215000???0?11021100011000?01---111---1-------110110111010110011105001030212200110020102?100111011?

Thalassodromeus

00020?10301122-1??-?1101?215000???0?11021100011000?01---111---1-------?????????????????????????????????????????????????????

Quetzalcoatlus

00021210501?22-1??-?1001?21??????????1020100011001001---111---1-------1021121????011001110500?1302?2300111020?????001120?1?

Zhejiangopterus

00020210001122-1??-?1100?210000???0??1?2?100011001001---111---1-------??21121110?011?011?05?0123?21?3001110?010201001?2011?

Azhdarcho

000??????0?????1??????????????????????????0001?00????---111---1-------1021121?????????1?1050????0????????10???????0011?????

Allkaruen (holotype+referred)

?????????0?1???????????????000?101010????100??000010?000??0?100???0?0?011?12???????????????????????????????????????????????

Allkaruen (braincase)

???????????1???????????????000?101010??????????????????????????????????????????????????????????????????????????????????????

**2.4 List of synapomorphies:** Unambiguous synapomorphies obtained in the parsimony analysis are listed below for the nodes present in the strict consensus tree. The strict consensus tree is included first with the node numbers used in the synapomorphy list. The synapomorphy list details synapomorphic characters for each node (arranged in phylogenetic order), indicating if the synapomorphy was present in all or some of the most parsimonious trees.


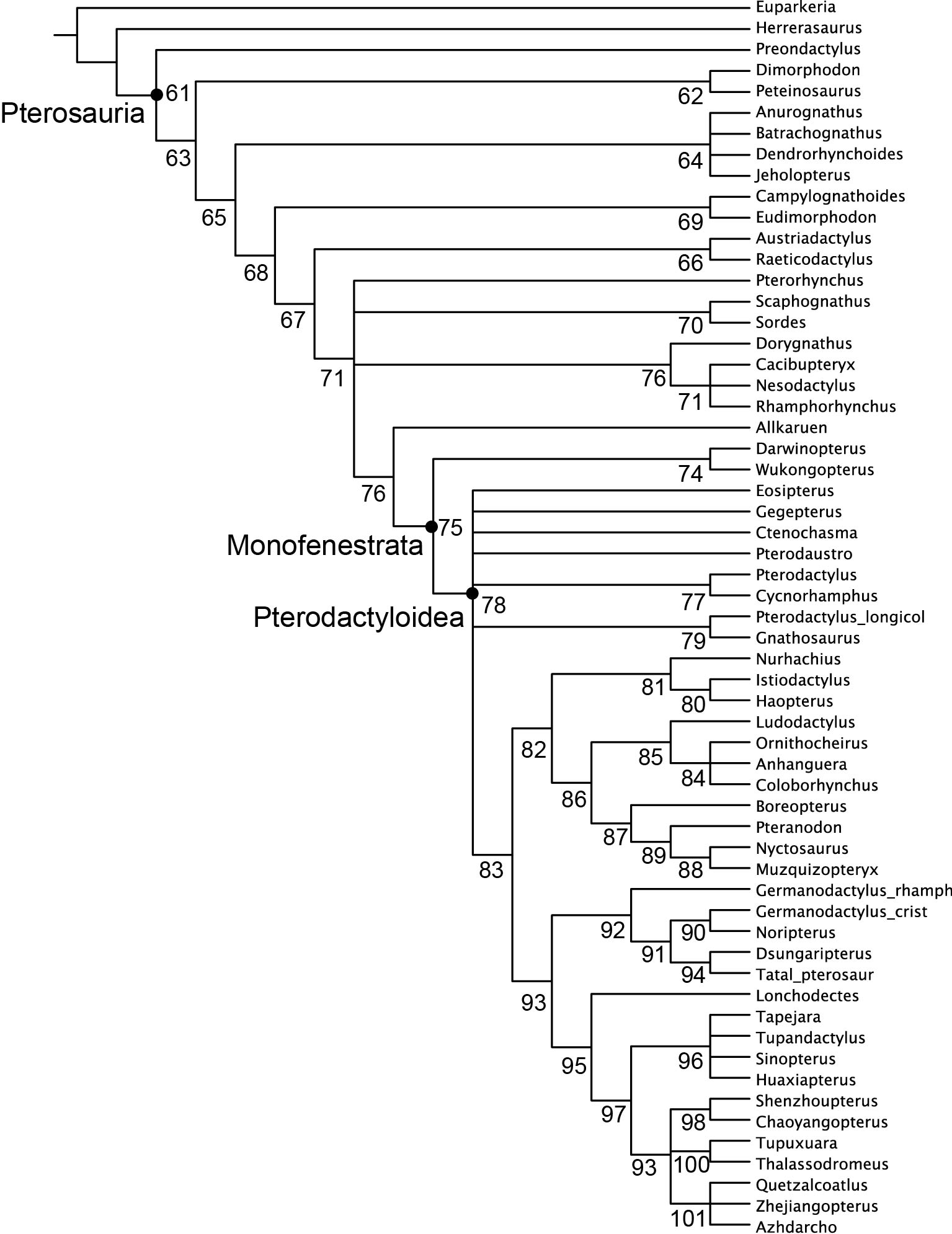


Unambiguous synapomorphies:

Node 61 :

All trees:

Char. 16: 0 --> 1

Char. 23: 1 --> 0

Char. 87: 0 --> 1

Char. 91: 0 --> 1

Char. 101: 0 --> 1

Char. 112: 0 --> 1

Char. 116: 1 --> 0

Node 62 :

All trees:

Char. 80: 0 --> 1

Node 63 :

All trees:

Char. 61: 0 --> 1

Char. 83: 0 --> 1

Char. 102: 0 --> 1

Node 64 :

All trees:

Char. 6: 1 --> 0

Char. 10: 0 --> 1

Char. 13: 1 --> 0

Char. 16: 1 --> 0

Char. 39: 0 --> 1

Char. 55: 0 --> 1

Char. 79: 0 --> 1

Some trees:

Char. 60: 0 --> 1

Char. 74: 0 --> 1

Char. 94: 0 --> 1

Char. 101: 1 --> 0

Char. 108: 0 --> 2

Char. 109: 1 --> 0

Node 65 :

All trees:

Char. 96: 0 --> 1

Char. 105: 0 --> 2

Char. 110: 0 --> 1

Node 66 :

All trees:

Char. 56: 0 --> 1

Some trees:

Char. 9: 0 --> 2

Node 67 :

All trees:

Char. 11: 0 --> 1

Char. 105: 2 --> 1

Node 68 :

All trees:

Char. 4: 0 --> 1

Char. 12: 0 --> 1

Char. 17: 0 --> 1

Char. 98: 0 --> 1

Char. 113: 0 --> 1

Char. 121: 0 --> 1

Node 69 :

All trees:

Char. 31: 0 --> 1

Char. 43: 0 --> 1

Char. 49: 0 --> 1

Char. 111: 0 --> 1

Node 70 :

All trees:

Char. 59: 0 --> 1

Some trees:

Char. 58: 0 --> 1

Node 71 :

All trees:

Char. 60: 0 --> 1

Char. 80: 0 --> 1

Char. 95: 0 --> 2

Char. 96: 1 --> 2

Some trees:

Char. 57: 0 --> 1

Char. 91: 3 --> 4

Node 72 :

Some trees:

Char. 20: 0 --> 1

Char. 23: 0 --> 1

Char. 103: 1 --> 2

Char. 107: 0 --> 1

Char. 109: 1 --> 0

Node 73 :

All trees:

Char. 15: 0 --> 1

Char. 48: 0 --> 1

Char. 51: 0 --> 2

Node 74 :

All trees:

Char. 105: 1 --> 0

Node 75 :

All trees:

Char. 33: 0 --> 1

Char. 71: 0 --> 1

Node 76 :

All trees:

Char. 36: 0 --> 1

Char. 73: 0 --> 1

Char. 75: 0 --> 1

Char. 76: 0 --> 2

Node 77 :

All trees:

Char. 71: 1 --> 0

Char. 72: 1 --> 0

Char. 98: 1 --> 2

Some trees:

Char. 7: 1 --> 0

Char. 88: 1 --> 2

Node 78 :

All trees:

Char. 74: 0 --> 1

Char. 78: 0 --> 1

Char. 79: 0 --> 1

Char. 80: 1 --> 0

Char. 101: 1 --> 2

Char. 109: 1 --> 0

Char. 122: 0 --> 1

Some trees:

Char. 47: 0 --> 1

Char. 88: 2 --> 1

Char. 95: 2 --> 1

Char. 108: 0 --> 1

Node 79 :

All trees:

Char. 2: 0 --> 1

Char. 44: 0 --> 1

Node 80 :

All trees:

Char. 6: 2 --> 1

Char. 99: 1 --> 0

Node 81 :

All trees:

Char. 3: 0 --> 1

Char. 45: 0 --> 1

Char. 70: 0 --> 1

Node 82 :

All trees:

Char. 76: 2 --> 1

Char. 77: 0 --> 1

Char. 83: 1 --> 2

Char. 84: 1 --> 0

Char. 86: 0 --> 1

Char. 91: 5 --> 7

Char. 92: 0 --> 1

Char. 93: 0 --> 1

Char. 97: 0 --> 1

Char. 102: 0 --> 1

Char. 105: 1 --> 2

Char. 115: 0 --> 1

Char. 119: 1 --> 2

Some trees:

Char. 40: 1 --> 2

Node 83 :

All trees:

Char. 94: 0 --> 1

Some trees:

Char. 100: 0 --> 1

Char. 120: 1 --> 0

Node 84 :

All trees:

Char. 9: 0 --> 1

Some trees:

Char. 52: 0 --> 1

Node 85 :

All trees:

Char. 2: 0 --> 1

Node 86 :

All trees:

Char. 22: 1 --> 0

Char. 23: 0 --> 1

Char. 26: 1 --> 0

Char. 65: 0 --> 1

Char. 82: 0 --> 1

Node 87 :

All trees:

Char. 5: 0 --> 1

Node 88 :

All trees:

Char. 24: 1 --> 0

Char. 84: 0 --> 1

Char. 91: 7 --> 8

Node 89 :

All trees:

Char. 28: 0 --> 2

Char. 50: 0 --> 1

Char. 57: 0 --> 1

Char. 58: 0 --> 1

Char. 59: 0 --> 1

Char. 63: 0 --> 1

Char. 100: 1 --> 2

Char. 101: 2 --> 3

Char. 105: 2 --> 3

Node 90 :

All trees:

Char. 120: 0 --> 1

Node 91 :

All trees:

Char. 22: 1 --> 0

Char. 67: 0 --> 1

Char. 87: 1 --> 0

Node 92 :

All trees:

Char. 9: 0 --> 2

Char. 11: 1 --> 0

Char. 35: 0 --> 1

Char. 64: 0 --> 1

Char. 66: 0 --> 1

Char. 76: 2 --> 0

Char. 111: 0 --> 2

Char. 116: 0 --> 1

Some trees:

Char. 7: 1 --> 0

Node 93 :

All trees:

Char. 60: 1 --> 0

Char. 81: 3 --> 1

Char. 114: 0 --> 1

Char. 117: 0 --> 1

Node 94 :

All trees:

Char. 28: 0 --> 3

Char. 53: 1 --> 0

Node 95 :

All trees:

Char. 72: 1 --> 0

Char. 89: 0 --> 1

Node 96 :

All trees:

Char. 1: 0 --> 1

Char. 52: 0 --> 1

Some trees:

Char. 9: 0 --> 4

Char. 28: 0 --> 34

Node 97 :

All trees:

Char. 57: 0 --> 1

Char. 58: 0 --> 1

Char. 59: 0 --> 1

Char. 63: 0 --> 1

Node 98 :

All trees:

Char. 8: 0 --> 1

Node 99 :

All trees:

Char. 100: 1 --> 2

Some trees:

Char. 40: 1 --> 2

Node 100 :

All trees:

Char. 9: 0 --> 3

Some trees:

Char. 41: 0 --> 1

Node 101 :

All trees:

Char. 106: 0 --> 1

Some trees:

Char. 73: 1 --> 2

Char. 101: 2 --> 3

Char. 119: 1 --> 2

**3. REFERENCES**

Bremer K. 1994. Branch support and tree stability. *Cladistics* 10:295–304.

Cúneo R, Ramezani J, Scasso R, Pol D, Escapa I, Zavattieri AM, Bowring SA. 2013. High-precision U-Pb geochronology and a new chronostratigraphy for the Cañadón Asfalto Basin, Chubut, central Patagonia: Implications for terrestrial faunal and floral evolution in Jurassic. *Gondwana Research*. DOI: 10.1016/j.gr.2013.01.010

Pol D, Rauhut OWM. 2012. [A Middle Jurassic abelisaurid from Patagonia and the early diversification of theropod dinosaurs](http://rspb.royalsocietypublishing.org/content/early/2012/05/17/rspb.2012.0660.short?rss=1). *Proceeding of Royal Society B: Biological Sciences* 279:3170–3175.
